# Molecular Genetics of Thrombotic Myeloproliferative Neoplasms: Implications in Precision Oncology

**DOI:** 10.3390/diagnostics13010163

**Published:** 2023-01-03

**Authors:** Yuh Cai Chia, Mat Jusoh Siti Asmaa, Marini Ramli, Peng Yeong Woon, Muhammad Farid Johan, Rosline Hassan, Md Asiful Islam

**Affiliations:** 1 Department Haematology, School of Medical Sciences, Universiti Sains Malaysia, Kubang Kerian 16150, Kelantan, Malaysia; 2 School of Health Sciences, Universiti Sains Malaysia, Kubang Kerian 16150, Kelantan, Malaysia; 3 Department of Molecular Biology and Human Genetics, Tzu Chi University, Hualien 97004, Taiwan; 4 Institute of Metabolism and Systems Research, University of Birmingham, Birmingham B15 2TT, UK

**Keywords:** essential thrombocytosis, gene, mutation, myeloproliferative neoplasms, polycythaemia vera, polymorphism, primary myelofibrosis, thrombosis

## Abstract

Classical *BCR-ABL*-negative myeloproliferative neoplasms (MPN) include polycythaemia vera, essential thrombocythaemia, and primary myelofibrosis. Unlike monogenic disorders, a more complicated series of genetic mutations are believed to be responsible for MPN with various degrees of thromboembolic and bleeding complications. Thrombosis is one of the early manifestations in patients with MPN. To date, the driver genes responsible for MPN include *JAK2*, *CALR*, *MPL*, *TET2*, *ASXL1*, and *MTHFR*. Affords have been done to elucidate these mutations and the incidence of thromboembolic events. Several lines of evidence indicate that mutations in *JAK2*, *MPL*, *TET2* and *ASXL1* gene and polymorphisms in several clotting factors (*GPIa*, *GPIIa*, and *GPIIIa*) are associated with the occurrence and prevalence of thrombosis in MPN patients. Some polymorphisms within *XRCC1*, *FBG*, *F2*, *F5*, *F7*, *F12*, *MMP9*, *HPA5*, *MTHFR*, *SDF-1*, *FAS*, *FASL*, *TERT*, *ACE*, and *TLR4* genes may also play a role in MPN manifestation. This review aims to provide an insightful overview on the genetic perspective of thrombotic complications in patients with MPN.

## 1. Introduction

The classical *BCR-ABL*-negative myeloproliferative neoplasms (MPN) of acquired clonal hematopoietic stem cell disorders include polycythaemia vera (PV), essential thrombocythaemia (ET) and primary myelofibrosis (PMF). In general, the signal-transduction pathways responsible for haematopoiesis are affected in these MPN [1,2]. PV is a condition in which over-proliferation of erythroid progenitors cause elevated red blood cell (RBC) mass and is usually accompanied by higher white blood cell (WBC) count, as well as an excessive number of platelets and bone marrow hypercellularity [3]. On the other hand, ET is a clonal haematological disorder arising from the proliferation of megakaryocytic lineage at an abnormal level resulting in increased platelet count [3,4]. Whereas PMF is characterized by the excessive proliferation of granulocytes, erythrocytes, and megakaryocytes associated with extensive bone marrow scarring and extramedullary haematopoiesis [5]. The 2016 WHO classification further classified PMF into pre-fibrotic (pre-PMF) and overt-fibrotic (overt-PMF) stages, defined from a classical morphological examination; fibrosis grade 0–1 in pre-PMF and 2–3 in overt-PMF [6]. Pre-PMF has distinct clinical features (anaemia, palpable splenomegaly, leukoerythroblastosis, leucocytosis, increased lactate dehydrogenase), disease outcomes, leukemic transformation, and survival rate. The verdict by the experts denoted pre-PMF as a unique combination of thrombo-hemorrhagic risk and a definite risk of disease evolution (to overt-PMF). Therefore, a correct differentiation of pre-PMF from PV or ET are crucial for the management of MPN [7]. 

The morphologic features on bone marrow trephine from PV, ET, and PMF patients are presented in Table 1. The most common causes of morbidity and mortality in patients with MPN are thromboembolism and haemorrhage [8,9]. Different types of thrombosis at different sites, including arterial and venous thrombosis, are observed in patients with MPN [10]. Thrombotic events are unequally distributed among MPN subtypes, and are notably more common in PV and ET patients but less frequent in PMF patients [11]. The incidence of thrombotic complications is about 28.6% 30.0%–41.0% in PV, 20.7% 19.0%–32.0% in ET, and 9.5% 7.2%–15.0% in PMF (Table 2) [12].

The role of genetic predisposition and landscape in the development of MPN have been quite extensively elucidated [13]. The discovery of driver genes *JAK2*, *CALR*, and *MPL* is an important milestone in unrevealing the myths of MPN, hence the World Health Organization has included mutations in all three genes as the hallmark of MPN [1]. Mutations in other genes, such as *TET2*, *ASXL1*, *DNMT3A*, *RUNX1*, *MIR662*, *EZH2*, *MLH1*, *MLH3*, *SF3B1*, *MSH6*, *MSH2*, *BARD1*, *KIT*, and *NRAS* [14], *SRSF2*, *IDH1* and *IDH2* [15], *TP53* [16], *F5 (G1691A)* and *F2* (*T165M)* [17], have also been reported. Aside from gene mutations, emerging evidence suggested that a numbers of single nucleotide polymorphisms (SNPs) are associated with the risk of thrombosis in MPN patients [18]. 

To the best of our knowledge, there is no overview on the genetic contributing factors in thrombotic MPN; therefore, this review aims to provide an insightful overview on the genetic and epigenetic perspective of thrombotic and bleeding complications in patients with MPN. Besides, the risk assessment of thrombosis based on the gene mutations, some possible pathogenesis of thrombotic MPN and current available drugs and therapies for thrombosis in MPN patients have been discussed in this review as well.

## 2. Gene Mutations in Thrombotic MPN

**Figure 1:**
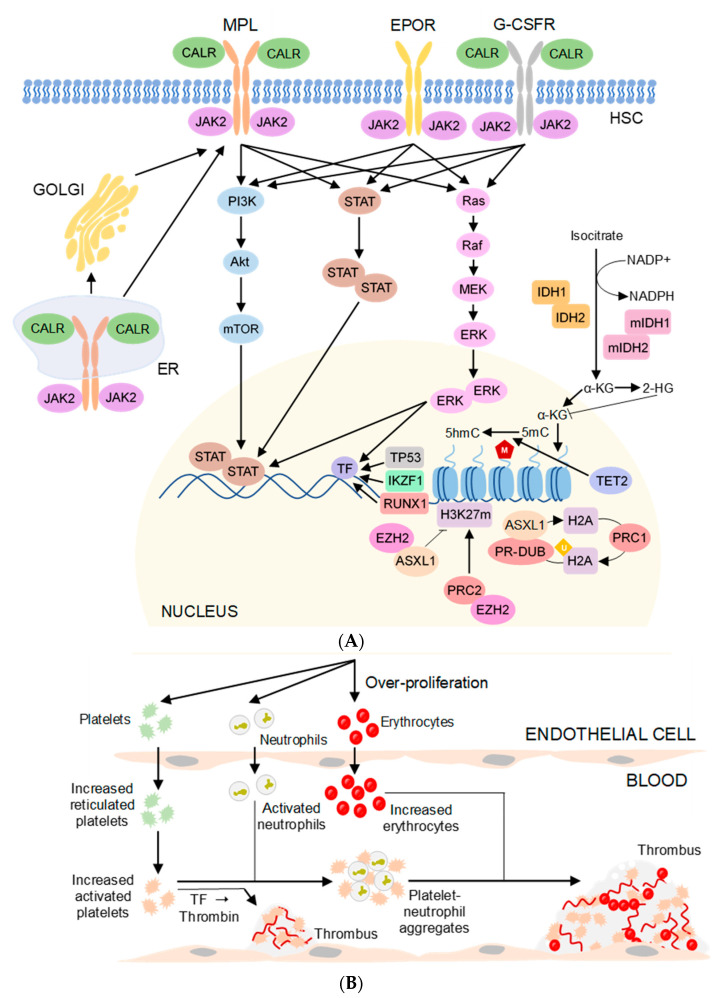
(**A**) Pathogenesis of thrombosis in MPN patients. Mutated JAK2, CALR, and MPL activate a JAK/STAT pathway, PI3K/Akt pathway, and MAPK/ERK pathway. Mutations in ASXL1 and TET2 cause epigenetic changes in DNA (ubiquitination, methylation and hydromethylation) and affect the differentiation of hematopoietic cells. All these lead to proliferation of blood cells at an abnormal rate. (**B**) The pathogenesis of thrombosis in MPN is complex and caused by multifactorial, other than the abnormal signalling transduction, platelet activation, endothelial cell dysfunction, over-production of tissue factors, formation of platelet-neutrophil aggregates, and increased red blood cell mass may further contribute to the genesis of thrombi. Most of the gene polymorphisms mentioned in this review take parts in initiating those abnormalities. Abbreviations: α-KG, α-ketoglutarate; 2-HG, 2-hydroxyglutarate; 5hmC, 5-hydroxymethylcytosine; 5mC, 5-methylcytosine; ASXL1, Additional sex combs like 1; Akt, Protein kinase B; CALR, Calrecticulin; EPOR, Erythropoietin receptor; ER, Endoplasmic reticulum; ERK, Extracellular signal regulated kinase; EZH2, Enhancer of zeste homolog 2; G-CSFR, Granulocyte colony stimulating factor receptor; HSC, Hematopoietic stem cell; IDH1, Isocitrate dehydrogenase 1; IDH2, Isocitrate dehydrogenase 2; IKZF1, Ikaros family zinc finger protein 1; JAK2, Janus kinase 2; MEK, Mitogen-activated protein kinase; MPL, Myeloproliferative leukaemia virus oncogene; mTOR, Mammalian target of rapamycin; NADP+, Nicotinamide adenine dinucleotide phosphate; NADPH, Nicotinamide adenine dinucleotide phosphate; PI3K, Phosphoinositide-3-kinase; PRC1, Polycomb repressive complex 1; PRC2, Polycomb repressive complex 2; PR-DUB, Polycomb repressive deubiquitinase; RUNX1, Runt-related transcription factor 1; STAT, Signal transducer and activator of transcription protein; TET2, Ten-eleven translocation 2; TF, Tissue factor; TP53, Tumour Protein p53.

**Figure 2:**
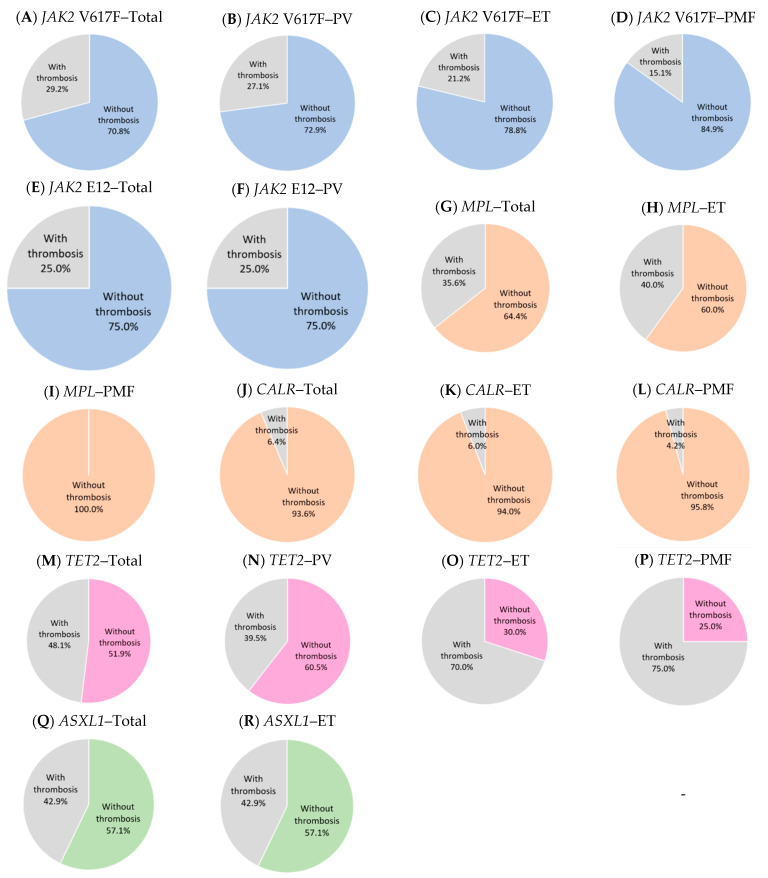
Thrombotic MPN patients with different gene mutations. (**A**–**F**) Tyrosine kinases. (**G**–**L**) Receptors. (**M**–**P**) DNA methylation. (**Q**,**R**) Histone methylation. Abbreviations: *ASXL1:* Additional Sex Combs Like 1, *CALR*: Calrecticulin, ET: Essential thrombocythaemia, *JAK2*: Janus kinase 2, PMF: Primary myelofibrosis, *MPL*: Myeloproliferative leukaemia virus oncogene, PV: Polycythaemia vera, *TET2*: Ten-eleven translocation 2.

**Figure 3:**
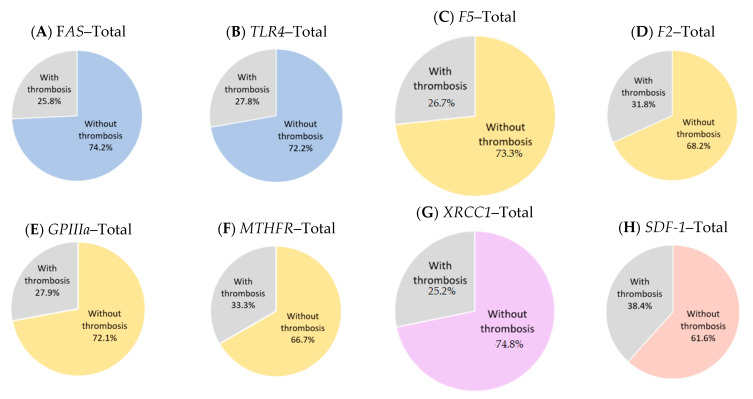
Thrombotic MPN patients with different gene polymorphisms. (**A**,**B**) Receptors. (**C**–**F**) Thrombophilic genetic factors. (**G**) DNA repair. (**H**) Chemokine. Abbreviations: *F2*: Factor II, *F5*: Factor 5, *FAS*: Fas cell surface death receptor, *GPIIIa*: Glycoprotein IIIa, *MTHFR*: Methylenetetrahydrofolate reductase, *SDF-1*: Stromal cell-derived factor-1, *TLR4*: Toll-like receptor 4, *XRCC1*: X-ray repair cross complementing 1.

The electronic searches identified all mutated genes in thrombotic MPN. However, only a few genes are subjected for further investigation; these are JAK2, CALR, MPL, TET2, and ASXL1. Several genes are involved in initiating signalling pathways, which play important roles in the pathogenesis of thrombosis in MPN (Figure 1A). In addition to the contribution of gene mutations (Figure 2), some SNPs (Figure 3) are associated with the risk of thrombotic manifestations in patients with MPN. The detail information for constructing the tables is presented in Table S1. A summary of the related gene mutations and SNPs are summarized in Table S2.

### 2.1. JAK2 V617F

*Janus kinase 2* (*JAK2*) located at chromosome 9p24.1 encodes a non-receptor tyrosine kinase involved in cell growth, differentiation, development, or histone modification. *JAK2* phosphorylates tyrosine within the cytoplasmic region of a cytokine receptor once ligands bind to type I or II receptors that are associated with the *JAK2* protein, thereby generating several docking sites for recruitment and phosphorylation of STAT proteins. Phosphorylated STAT proteins dimerized in cytoplasm and transport into the nucleus for further gene activations [19]. In 2005, the *JAK2* V617F was identified as one of the molecular markers of MPN [20,21]. The *JAK2 V617F* have been detected in 46.7 to 100% in patients with PV, from 31.3 to 72.1% in patients with ET, and from 25.0 to 85.7% in those with PMF (data from 2000–2018) [22]. The *JAK2* gene mutations have been detected in approximately 95% of patients with PV, up to 70% of patients with ET, and 40–50% of patients with PMF [23,24]. One of the most common types of mutation of *JAK2* is the V617F, a somatic gain-of-function mutation that changes the 1849th coding nucleotide from guanine (G) to thymine (T) resulting in a replacement of amino acid from valine to phenylalanine (c.1849G > T, p. Val617Phe), which is strongly associated with MPN. Accumulative evidences suggested that the V617F mutation increased the risk of thrombosis [25,26,27,28,29] and could be considered as a predictive biomarker of thrombotic events in MPN [30,31,32]. Thrombosis is significantly and frequently found in patients with V617F mutation (30.2%) than without (9.2%, *p* = 0.04). Patients with *JAK2* V617F-positive MPN are older in age (*p* = 0.003) and displayed high levels of haemoglobin (*p* < 0.01) and haematocrit (*p* < 0.01) and low levels of erythropoietin (*p* < 0.01). In addition, the same group of patients exhibited a three-fold increase in leucocytosis and a two-fold increase in thrombosis and splenomegaly and correlate well with high WBC counts of >10,000/μL (*p* = 0.046). Therefore, the *JAK2* V617F mutation should be determined in patients with MPN especially in those aged 60 years or older and with a history of previous thrombosis and leucocytosis [33,34,35]. On the other hand, MPN-associated thrombosis is very rare in children and adolescents. To date, thrombosis was observed in three cases of young patients with MPN with a positive *JAK2* driver mutation. Similar observation was obtained from another study, where 17 out of 19 children with MPN have thrombosis (89.5%) and the same driver mutation. It is therefore speculated that children with MPN may have higher thrombotic risk when driver mutation is present [36]. In a cohort study from Czech, the *JAK2* mutation was detected in 145 patients with MPN, 40 of whom had thrombosis (27.6%), which was significantly higher than those without (8.0%, *p* = 0.001). Among 78 patients with thrombosis, 20 (25.6%) had a positive thrombophilia status (a pro-thrombotic state or hypercoagulability), and only 48 cases were identified in patients without thrombosis (14.0%, *p* = 0.016). Hence, the *JAK2* mutation and the presence of additional thrombophilic markers, such as thrombophilia status, predispose patients with MPN with thrombocythaemia to thrombosis [37]. In another cohort study from Hungary [38], they reported that the V617F mutation statistically had no predictive value for the development of thrombosis in patients with MPN.

In another cohort study from Taiwan, the V617F mutation was frequently detected in patients with MPN (*p* = 0.007) with higher haemoglobin level (*p* = 0.03) and WBC count (*p* = 0.002), splenomegaly (*p* = 0.01), and longer disease duration (*p* = 0.005), but with no obvious difference on the overall thrombosis risk (*p* = 0.22). Amongst Mexican mestizos, the cause of thrombosis may not necessarily be related to MPN. Therefore, testing for the JAK2 V617F mutation was not mandatory in patients with unexplained thrombophilia in the country except for those who had thrombosis in uncommon sites or having abnormal cell counts suggestive of MPN [39]. Thus, screening for the JAK2 mutation in patients with BCR-ABL-negative MPN could be significant in identifying patients with a high chance of developing vascular complications [40]. In a study on patients with MPN (n = 391), JAK2 V617F mutation-positive subjects were more susceptible to thrombotic events than JAK2 V617F-negative subjects, which is statistically significant among ET (*p* < 0.01) and PMF groups (*p* < 0.01) [41]. Although the mutation is related to the risk of thrombosis in ET, it has no relationship with recurrent thrombosis in ET [42]. In other studies, on patients with ET, patients with the JAK2 mutation exhibited significantly more frequent thrombotic events (*p* = 0.004), higher haemoglobin levels (*p* = 0.0003), and leukocyte counts (*p* < 0.0001) [35,43,44,45].

Patients with *JAK2*-positive ET consistently display *JAK2* activation, which in turn upregulates the expression of the heparanase enzyme via the erythropoietin receptor activation [46]. The *JAK2* V617F mutation as a thrombotic risk factor in ET may also have a role in causing increased myeloid proliferation and activation of WBCs. According to Patriarca et al. [47], the *JAK2* V617F mutation was significantly related to thrombotic events before (*p* < 0.0003) or during diagnosis (*p* < 0.03) and had no protective role in haemorrhagic risk. The *JAK2* V617F mutation was also related to the overexpression of polycythaemia rubra vera 1 (*p* < 0.0001), higher haematocrit (*p* < 0.03), lower platelet count (*p* < 0.0006), and higher WBC count (*p* < 0.0002) with >8.4 × 109/L, which was found to be significantly related to increased thrombosis risk (*p* < 0.006). The number of megakaryocytes increased significantly (*p =* 0.031), and hypercoagulable status was observed (*p =* 0.038) in patients with *JAK2* V617F-positive PV and ET. Many studies suggested that the *JAK2* V617F mutation may play a part in blood coagulation by modifying the number and functions of RBCs, WBCs, and platelets in patients with MPN [48,49,50,51].

Patients with PV with the *JAK2* V617F allele burden of >75% exhibited higher thrombotic risk than those with <25% (*p* = 0.03) [52] and higher risk of venous thromboembolism (VTE) when their *JAK2* allele burden is >20%, but not for arterial thrombosis [53]. The risk of VTE was increased by 7.4-fold when the *JAK2* allele burden is >20% (*p* = 0.004), 8.8-fold by 50% (*p* = 0.006) and there is a more significant risk with allele burden of >75% (*p* = 0.002), with mostly cases with proximal deep vein thrombosis (DVT) (*p* = 0.041) for allele burden of >50%. Therefore, the allele burden of *JAK2* V617F is related to the risk of VTE [53]. Patients with PMF with *JAK2* V617F allele burden of ≤34.8% were more susceptible to thrombosis (*p* = 0.032) [54]. The prevalence of arterial thrombosis increased in patients with *JAK2*-mutated ET, but no association was found between the allele burden and thrombosis (*p* = 0.001) [55]. The allele burden of *JAK2* V617F is related to the clinicohaematological phenotypes of patients with ET, such as older age (*p* = 0.03), organomegaly (*p* = 0.003), higher neutrophil count (*p* = 0.02), thrombotic events (*p* = 0.01), and myelofibrosis (*p* = 0.02) in ET, but a larger scale of studies is needed for patients with PV and PMF [28,45]. Another study reported that thrombotic events were frequent in Korean patients with the *JAK2* V617F mutation (*p* = 0.045), especially those with the homozygous *JAK2* V617F compared with heterozygotes and wild type (*p* = 0.03) [56]. Since the *JAK2* V617F allele burden could only predict the thrombosis sites and not thrombosis timing, screening for *JAK2* V617F would be of great help for MPN to identify any underlying vascular problems [57].

An underlying MPN may present in patients with splanchnic vein thrombosis (SVT) with *JAK2* V617F mutation. Most patients with SVT carried a *JAK2* V617F mutation, which was associated with higher leukocyte and platelet counts and higher mean values for levels of lactate dehydrogenase (*p* = 0.08). There was no correlation between *JAK2* V617F and pro-thrombotic risk factors and sites of thrombosis. The researchers suggested that the testing of *JAK2* V617F may help in the discovery of latent MPN in patients with SVT [58,59,60,61,62], but not in VTE, due to its weak association with *JAK2* mutation [63]. A different outcome, as indicated in other studies [53,64], showed that patients with MPN with *JAK2* V617F, in fact, had a higher risk of VTE (*p* = 0.024), especially of DVT. Routine tests for venous thrombosis were recommended in patients with MPN, especially when the patients exhibited abdominal pain and increasing pruritus. This is because individuals with thrombosis are usually accompanied by a higher prevalence of pruritus (*p* = 0.02) and abdominal pain (*p* = 0.03). In contrast, patients with venous thrombosis, particularly of the portal vein or unusual sites, should also be screened for MPN [65]. SVT comprises portal vein thrombosis (PVT) and Budd-Chiari syndrome (BCS), which are the common first presenting symptoms in latent MPN [66,67,68]. There was no significant difference between BCS and PVT for the prevalence of the *JAK2* V617F mutation (*p =* 0.989), except that a significantly higher female predominance (*p* < 0.001), younger age of thrombosis diagnosis (*p =* 0.007) [69], and higher platelet counts (*p =* 0.004) were observed in BCS with *JAK2* V617F. As for *JAK2* V617F-positive PVT, the number of WBC (*p =* 0.003) and platelet counts (*p =* 0.01) seemed to be higher [58]. Other than PVT, testing for *JAK2* V617F was also recommended for patients with idiopathic hepatic vein thrombosis [58] and with extrahepatic PVT for the early identification of latent MPN [70,71]. In a China cohort study [72], a lower frequency of *JAK2* V617F mutation was found in Chinese patients with BCS, suggesting that the major cause of BCS in this population might not be MPN, whereas the *JAK2* V617F mutation indicated a higher prevalence in non-cirrhotic and non-malignant PVT patients (*p* < 0.001). Thus, routine screening was suggested for these two groups of PVT patients. However, an India cohort study indicated that the *JAK2* V617F mutation was found at a very low frequency and displayed a weak association with thrombosis, particularly for venous thrombosis present in sites other than the splanchnic region [73]. A low incidence of the *JAK2* V617F mutation was observed in Korean patients with SVT [74]. However, a screening test is not feasible due to the very low frequency of *JAK2* V617F mutation (rs77375493) in general population, the minor allele T frequency is about 0.016% in East Asian according to 1000 Genome Database. In addition, Asian countries displayed a greater genetic heterogeneity among Asian populations [75].

Of 22 patients, 9 (41.0%) with normal blood counts and major intra-abdominal vein thrombosis exhibited the *JAK2* V617F mutation, which is in line with the results of patients with SVT, where 39% of the patients were found to have the same mutation [69]. Hence, to detect any latent MPN, screening for the *JAK2* V617F mutation was recommended in patients with major idiopathic abdominal vein thrombosis who have normal blood counts [76], but not in patients with arterial and venous thrombosis at usual sites [77]. A study was also conducted in patients with arterial thrombosis for *JAK2* V617F mutation and no mutation was found in any of the patients. Therefore, it was concluded that testing for the *JAK2* V617F mutation in those patients is not compulsory, even in young patients with relatively high peripheral blood counts [78]. As for other types of thrombosis, *JAK2* V617F can present in patients with cerebral venous thrombosis irrespective of blood count (*p* < 0.0001) and as one of the early symptoms of MPN [79]. Despite this, genetic testing in unselected cerebrovascular cases was not recommended because the *JAK2* mutation is low [77], but genetic testing can be considered in patients with embolic strokes caused by cerebral vein thrombosis, especially those with haemoglobin levels >16.5 g/dL or a platelet count >450,000/mm^3^ [80]. According to Linnemann et al. [81], the *JAK2* V617F mutation or the presence of MPN is not associated with inferior vena cava thrombosis (IVCT). The common risk factors for IVCT are previous surgery, hormonal therapy in women, hereditary thrombophilia, and cancers other than MPN. High occurrence of the *JAK2* V617F mutation was present in patients with intra-abdominal venous thrombosis (IAVT). Therefore, screening for *JAK2* V617F mutation for latent MPN was suggested for IAVT patients [82]. Mesenteric vein thrombosis can occur in patients with normal peripheral blood counts and may be one of the first presenting features of MPN. Approximately 33% of patients with MVT were detected with the *JAK2* V617F mutation and were confirmed to have MPN [83]. Screening for *JAK2* V617F was also suggested in patients with idiopathic chronic portal, splenic, and mesenteric venous thrombosis, owing to its high prevalence [84].

Taken together, we can conclude that *JAK2* V617F mutation is strongly associated with the occurrence of thrombosis in patients with MPN. Therefore, genetic testing of *JAK2* V617F mutation among MPN patients could be beneficial in identifying any latent MPN in patients with different types of thrombosis.

### 2.2. JAK2 Exon 12

PV patients frequently displayed *JAK2* exon 12 mutations, with either a low prevalence or none in ET or PMF [85,86,87,88]. Interestingly, its prevalence was detected to be high (up to 40%) when investigated in *JAK2* V617F-negative patients [89,90,91,92,93]. In some cases, *JAK2* exon 12 mutation coexists with the *JAK2* V617F mutation [94]. According to a study of 106 patients with *JAK2* exon 12-mutated PV, the most common type of mutation N542-E543del was detected in 30% of patients. Patients with the mutated *JAK2* exon 12 were also having significantly higher haemoglobin levels (*p* < 0.001) and lower platelet (*p* < 0.001) and leukocyte counts (*p* < 0.001), but a similar thrombotic risk (*p* = 0.40) to those with *JAK2* V617F-mutated PV [95]. Unlike the *JAK2* V617F mutation, patients with SVT exhibited very low association with the *JAK2* exon 12 mutation; therefore, screening for the *JAK2* exon 12 mutation in patients with SVT is not recommended [96]. To date, only one study reported that patients with PV with mutated *JAK2* exon 12 have the same risk of thrombosis as those with the *JAK2* V617F mutation. Since only limited data indicated the association between *JAK2* exon 12 mutation and MPN, more studies are needed to have a better understanding of the *JAK2* exon 12 mutation.

### 2.3. CALR

*Calreticulin* (*CALR*) located at chromosome 19p13.13 is a calcium-binding chaperone that regulates calcium signalling, promotes protein folding and assembly of oligomers, and participates in the quality control system in the endoplasmic reticulum through calnexin/*CALR* cycles [97]. It also travelled to the nucleus, implying that it may play a crucial role in the regulation of gene transcription [98].

*CALR* mutation was found to be more prevalent in patients with ET and PMF than patients with PV [99,100]. Patients with positive *CALR* mutation significantly involved a younger age population (ET, *p* = 0.025; PMF, *p* = 0.002) and their platelet counts were higher than *JAK2* mutation-positive subjects (ET, *p* < 0.001; PMF, *p* = 0.001). Interestingly, a remarkably less frequent (*p* = 0.03) venous thrombosis was observed in patients with ET with *CALR* mutation than those with *JAK2* mutation [35]. Other coagulation complications, such as arterial thrombosis (14% vs. 9%; *p* = 0.3) and haemorrhage (9% vs. 5%; *p* = 0.3), were not significantly different in patients with ET with *JAK2* mutations than those with *CALR* mutations. In another cohort of Italian patients [101], the risk of developing thrombosis was 2.5-fold higher in patients with ET (7.1% vs. 2.8%; *p* = 0.059), and 3.7-fold higher in patients with PV (10.5% vs. 2.8%; *p* = 0.001) with *JAK2* mutations compared with *CALR* mutation. Similar results were observed by Rotunno et al. [102], who found that patients with ET exhibiting *CALR* mutations had significantly (*p* = 0.01) lower risk of developing thrombotic events (13.5%) than those with *JAK2* (30.1%) and *MPL* mutations (40.0%).

In a French cohort study [103], 81% of patients with SVT had the *JAK2* V617F mutation, whereas only 1.6% had *CALR* mutation, same with the study outcome in a Spanish cohort [104], where only 1.9% of patients with SVT (n = 209) were found to have the *CALR* mutation. The *CALR* mutation is found to activate the thrombopoietin (TPO) receptor, *MPL*, and results in the expansion of the megakaryocytic lineage, which subsequently increases the platelet counts and rate of splenic enlargement in MPN [105]. *CALR* mutation is mutually exclusive to the *JAK2* V617F in patients with SVT; therefore, it is suggested that *JAK2* V617F should be tested first in this group of patients. Once ruled out of the *JAK2* V617F mutation, *CALR* mutation should then be investigated. However, screening for *CALR* mutations should be considered when patients presented a spleen height of >16 cm and platelet count of >200 × 109/L [103]. It was also suggested to test for *CALR* gene mutation in patients with MPN for prevention and treatment of SVT [106].

### 2.4. MPL

Myeloproliferative leukaemia virus proto-oncogene (*MPL*) is located at chromosome 1p34.2. The encoded protein acts as a TPO receptor that regulates megakaryopoiesis through the JAK-STAT pathway and platelet production. MPL also plays a role in immune response [107,108]. *MPL* mutations cause cytokine-independent growth and increased sensitivity towards TPO leading to the constitutive activation of downstream signalling pathways, namely JAK-STAT, PI3K-Akt and MAPK/ERK pathways [109,110].

A study reported that *MPL* mutations were only found in ET and patients with PMF, but not in patients with PV, chronic myeloid leukaemia, myelodysplastic syndrome or acute myeloid leukaemia [111,112]. In patients with ET, the burden of W515K allele was higher than W515L allele, although the difference between these two mutations in the alteration of signalling remains ambiguous. More than 50% allele burden in the peripheral blood was not found in all patients with ET; however, it was observed in most of the patients with PMF, indicating that patients with PMF may possibly be in an advanced stage of a previously undiagnosed MPN [113]. An *MPL* mutation screening study on 1182 patients with MPN [112] found that *MPL* mutations were present in 1% of patients with ET and in 5% of patients with PMF. Six patients were concurrently identified to have both *JAK2* and *MPL* mutations, suggesting a possibility of functional complementation in MPN diseases. Rotunno et al. [102] observed that patients with *MPL*-positive mutations displayed a significantly low level of haemoglobin (*p* < 0.001) and considerably higher platelet counts (*p* = 0.006) and serum erythropoietin (*p* < 0.001) than those with *JAK2*-positive ET. There were no obvious differences in the frequency of thrombosis, fibrotic transformation, major haemorrhage, or mortality rate in patients with *MPL* mutations compared with *JAK2*-positive, *JAK2*-negative and *MPL*-negative groups. However, in terms of the frequency of venous thrombosis, *MPL* patients had a significantly higher rate than *JAK2*-negative patients (*p* = 0.02) in addition to exhibiting microvascular symptoms in MPL patients compared with other mutations (*p* < 0.01). According to the findings of a PT-1 study [114], amongst 776 patients with ET, 4.1% of patients were found to have *MPL* mutations, where 1 patient had co-occurrence of *JAK2* mutation. No correlation was established between *MPL* mutations and thrombosis, major haemorrhage, and fibrotic transformation. According to Akpına et al. [115], no *MPL* mutations were found in patients with ET. Of 77 patients, 18.2% of patients with PMF presented with *MPL* mutations but without any thrombotic or haemorrhagic complications. Based on the existing evidence, *MPL* mutations seem to be not related to the development of arterial thrombosis. However, these mutations may have a relationship with venous thrombotic events in patients with MPN.

### 2.5. TET2

*Ten-Eleven Translocation 2* (*TET2*) gene is located at chromosome 4q24, a region wherein recurrent microdeletions, translocations, and uniparental disomy occurred frequently in patients with myeloid malignancies [116,117]. Upon phosphorylation and activation by cytokine-stimulated *JAK2*, *TET2* speeds up the conversion of 5-methylcytosines to 5-hydroxymethylcytosines, which is an essential step for the differentiation of hematopoietic stem cells into erythroid cells [118,119]. According to several in vivo and in vitro studies [117,120,121], mutated *TET2* causes myeloid proliferation and development of myeloid malignancies, which further suggests that *TET2* may act as a tumour suppressor in maintaining the homeostasis of hematopoietic cells [122].

*TET2* mutations significantly clustered in older aged patients (*p* < 0.0001) [123]. The mutation cluster mainly distributed on exons 3 to 11 with no typical hot spot mutation. Small insertions, nonsense mutations and small deletions gave rise to the most common loss-of-function mutations in *TET2* [123,124]. According to Ha et al. [122], the mean frequency of *TET2* mutation was 12.1% and displayed no significantly difference among PV (22.2%), ET (9.7%), PMF (18.2%), and unclassified MPN (0%) (*p* = 0.314). No relationship was detected between *TET2* mutations and thrombosis in PV (*p* = 0.446), ET (*p* = 0.325) and PMF (*p* = 0.182) [122]. Recently, Segura-Díaz et al. [125] conducted an interesting study on 68 patients with MPN (16 PV, 25 ET, 16 PMF and 11 secondary myelofibrosis), wherein thrombotic events were observed in 32.4% of the patients. In this cohort, the most frequently observed mutation was with the *TET2* gene (32.6%), besides *ASXL1* (14.0%) and *DNMT3A* (14.0%) genes (DTA genes). Pathogenic DTA gene mutations (*p* = 0.02), especially *TET2* gene mutation (*p* = 0.03), were found to be significantly accompanied by the development of thrombosis in the PV group. In a study conducted in Italy [124], novel *TET2* mutations were identified in 3 of 23 patients with SVT. They had no other inherited or acquired thrombophilic risk factors. Of the three patients, one had overt MPN and two were diagnosed with MPN years after their venous thrombosis. After studying both *TET2* and *JAK2* mutations, it was found that *TET2* is usually related to acquisition at the early event, whereas *JAK2* is a subsequent acquisition in MPN [124]. On the contrary, another study predicted that *TET2* might preferably happen after *JAK2*; therefore, TET2 is more likely to contribute to illness progression, such as thrombosis in patients rather than disease development [122] and *TET2* mutations can be screened to identify the patients to whom more attention should be paid for any subsequent development of overt disease [124].

Taken together, *TET2* mutation may increase the risk of thrombosis in patients with PV. However, larger sample size studies, including a large *TET2*-mutated cohort, should be conducted to obtain a comprehensive insight.

### 2.6. ASXL1

Additional sex combs-like 1 (*ASXL1*) gene is located at chromosome 20q11.21 and the encoded chromatin protein functions in maintaining the stable activation and silencing of mainly homeotic loci. In addition, this protein is also involved in genetic and epigenetic regulations and transcription control of several genes [126,127,128]. Many different types of mutations have been detected in exons 12 and 13 of the *ASXL1* gene; amongst them, the most common mutation is the c.1934dupG frameshift mutation, which is composed of 50% of all *ASXL1* mutations detected [129,130]. Amongst patients with MPN, *ASXL1* mutations were frequently found in patients with advanced age and in patients with PMF and post-PV/ET MF patients than those with PV or ET [131,132,133]. The frequencies of *ASXL1* mutations in PV, ET, and PMF were 3.5%, 5.6%, and approximately 23%, respectively [129,130,134]. In a Chinese cohort study [135], *ASXL1* mutation was detected in 19.4% of patients with ET, where missense mutation (c.G1954A) was the most common mutation type. The frameshift mutation (c.1934dupG) was not detected in this cohort. Patients with *ASXL1*-mutated ET exhibited to develop significantly higher thrombotic events (*p* = 0.021) than *ASXL1* wild type. In patients with PMF, *ASXL1* mutations were found to be significantly related with leucocytosis (*p* < 0.001 in a European cohort and *p* = 0.007 in a Mayo clinic cohort) in addition to being associated with significantly shorter overall survival (*p* < 0.0001) [136,137].

ET patients harbouring the *ASXL1*-mutation are prone to experience thrombotic events, it can be postulated that *ASXL1* mutations may partake in the occurrence of thrombohaemorrhagic events in ET. Although the relationship between *ASXL1* mutations and the thrombotic pathogenesis of ET remains unclear, one study suggested that one of the potential strategies to prevent thrombotic events in patients with ET is through blocking the *ASXL1* mutations [135]. Therefore, future studies are required to assess and evaluate the genetic contribution of ET in thrombosis.

**Table 2:** Molecular characteristics of PV, ET and PMF.

	PV [138]	ET [138]	PMF [139]
Prevalence of MPN	49.2%	34.7%	14.4%
Driver mutations			
*JAK2*	98%	52%	62%
*CALR*	0%	26%	22%
*MPL*	0%	4%	5%
Co-occurring mutations			
^‡^ *TET2*	22%	16%	15%
^‡^ *ASXL1*	12%	11%	48%
* Prevalence of thrombotic at diagnosis	28.6%	20.7%	9.5%
* Prevalence of bleeding at diagnosis	6.9%	7.3%	8.9%

* The pooled prevalence of overall thrombotic and bleeding events at diagnosis of MPN from 29 cohort studies [12]. ^‡^ The most frequent co-occurrence mutation in MPN [138,139].

## 3. Triple-Negative MPN

The principal driver mutations in MPN are constituted by *JAK2*, *CALR*, and *MPL* gene mutations, however, there is a sub-group in which none of the driver mutations is observed, the group is therefore called “triple-negative” MPN. Triple-negative MPN patients appear to have a relatively poor prognosis [140], higher rate of leukaemic transformation [141], and worse leukaemia-free survival [142]. The distribution of triple-negative cases in classical *BCR*-*ABL*-negative MPN is <1% in PV [143], 14% to 32% in ET, and 10% to 35% in PMF [144].

Triple-negative ET patients are usually younger with a higher male predominance, higher platelet count, and lower leukocyte counts and haemoglobin level [145]. The same group of patients show similar low rates of thrombosis as *CALR* mutated ET patients [102,146], and confer a way lower thrombotic risk compared to *JAK2* mutations. In both univariate (*p* = 0.009) and multivariate analysis (*p* = 0.003), in which the age (*p* = 0.01) and thrombosis history (*p* = 0.0006) are included, triple-negative ET patients display better thrombosis-free survival rate [147]. However, it is important to note that it is not necessarily the presence of triple-negative status that is related to lower thrombosis risk, but the absence of *JAK2* mutations [102].

So far, limited data is available for triple-negative PMF group compared to other PMF groups, triple-negative PMF shows a higher incidence of acute leukaemic transformation in triple-negative cases (*p* = 0.038) [35], and lower vascular risk [145].

## 4. Gene Polymorphisms in Thrombotic MPN

According to Afshar-Kharghan et al. [148] patients with PV exhibited a significantly higher overall frequency of arterial and venous thrombotic events (26% vs. 58%; *p* < 0.05) than patients with ET. Among patients with PV, a higher risk of arterial thrombosis (*p* < 0.05) was observed when the patients carried the PIA2 allele (*GPIIIa*, p.P33L), this polymorphism alters the protein conformation and leads to thrombotic complications. However, HPA-1a/1b GPIIIa variant failed to show any thrombosis correlation between PV and ET patients [149]. Study on another *GPIa/IIa* c.807C > T polymorphism [150], MPN patients with arterial thrombosis exhibited a higher frequency of TT genotype (26.5 vs. 11.5%, *p* = 0.049; odds ratio 2.68). Genetic predisposition may be stacked up in patients with MPN when SNPs affecting DNA repair and apoptosis are present. A polymorphism in the DNA repair gene *XRCC1* (Gln399Arg; rs25487) affects the base excision repair pathway, which may eventually lead to thrombotic and bleeding complications seen in patients with MPN (*p* = 0.386) [151]. Another gene polymorphisms within the *β-fibrinogen* (*FBG*), the heterozygous CT genotype of *FBG* c.-148C > T polymorphism is crucially higher (*p* = 0.02) in MPN patients with thrombotic events (57.7%) than CC wild type (40.0%) or TT homozygous (12.5%), but not for −455G/A *FBG* polymorphism [149]. Moreover, the presence of polymorphism in clotting factor II (F2) or G20210A mutation of prothrombin and clotting factor V (F5) or Leiden mutation has no significant association with thrombosis in patients with PV and ET [150,152]. A different outcome was obtained from another study [153], which found no difference between *F5* mutation and arterial thrombosis (*p* = 0.337), but a higher rate of detection of venous thrombosis before and at time of diagnosis and recurrence of venous thrombosis in patients with PV and ET (*p* = 0.03). It was also stated that F5 may increase the risk of thrombosis in patients with MPN [73,154]. As for coagulation factor, factor VII (F7), c.-323P10 variant was more frequently present in thrombotic patients with MPN (*p* = 0.04). In addition, the possibility of developing arterial thrombosis increases when both heterozygous *FBG* c.-148C > T and *F7* c.-323P0/10 SNP coexisted at the same time (*p* = 0.008). Hence, it was suggested that coexistence of different polymorphisms may contribute to the pathophysiology of thrombosis in MPN [150]. Another clotting factor, factor XII (F12), with −46C/T *F12* polymorphism did not correlate with thrombotic complication in patients with PV and ET [149]. In another study, decanucleotide insertion polymorphism of the *F7* gene significantly appeared as an independent risk factor in thrombotic development of overall patients with MPN (*p* = 0.0007), including patients with ET (*p* = 0.0002) [111]. 

Matrix metalloproteinases (*MMP9*) gene Gln279Arg A > G polymorphism was found in PV, ET, and secondary polycythaemia. Compared with controls, the incidence of *MMP9* polymorphism in patients with MPN was much higher (*p* < 0.05). A positive correlation was found between thrombosis, *JAK2* mutation (*p* = 0.006), and *MMP9* polymorphism (*p* = 0.002) in the ET group. Therefore, *MMP9* gene polymorphisms might be a contributing factor for the occurrence of vascular events in patients with MPN; however, more studies are warranted [155]. In the case of human platelet antigen (*HPA5*), a significant difference was observed in the genotype frequency between patients with MPN with and without vascular events (*p* = 0.03) with a protective role of *HPA5* b allele for patients with MPN [156]. In an interesting study, methylenetetrahydrofolate reductase (*MTHFR*) C677T was statistically significant in thrombotic patients with MPN compared with the general thrombosis population by both univariate (*p* = 0.001) and multiple regression analyses (*p* = 0.01). This finding anticipated that *MTHFR* polymorphism could have a role as a pro-thrombotic factor, causing thrombosis in patients with MPN [157]. Compared with controls, stromal cell-derived factor-1 (*SDF-1*) polymorphisms distributed significantly differently in patients with PV (*p* = 0.0003) and ET (*p* = 0.039). A more abundant amount of homozygous AA genotype was noted in PV (16%) and ET (11%) than in the control group (2%). Allele homozygous patients with PV (71%) and ET (67%) were significantly more common to have thrombosis than heterozygous and wild type groups (*p* = 0.03). Thus, the AA genotype can be considered as a predictor for thrombotic events in patients with PV and ET [158]. 

The FAS/FASL pathway is critical for hematopoietic cell survival and apoptosis [159,160]. Elevated *FAS*-670AG + GG distribution was reported in patients with MPN (*p* = 0.003), with A allele more common in both normal patients and patients with MPN and more frequent AG genotype detected in patients with MPN. However, no significant association was found between *FAS* 670A > G polymorphisms and venous thrombosis (*p* = 0.412) or splenomegaly (*p* = 0.08). For *FASL* 843C > T polymorphism, no difference was observed in either the genotype or allele frequency between patients with MPN and the control group (*p* = 0.144). No statistically significant correlation was also detected in venous thrombosis and splenomegaly. It was further suggested that *FAS* and *FASL* gene expression may have a role in the pathogenesis of MPN, but not possibly with thrombosis, yet more studies are needed to prove this finding [161]. 

The frequency of telomerase reverse transcriptase rs2736100_C variant was much higher in patients with MPN (*p* < 0.0001) regardless of the types of disease or molecular background, although this *TERT* variant was not associated with thrombosis [162]. In patients with *JAK2* V617F-positive PV and ET, angiotensin-converting enzyme (ACE), II genotype (*p* = 0.009), and I allele frequency (*p* = 0.004) were substantially higher than the control group. Patients tend to present a lower risk for MPN with DD genotype (*p* = 0.02) and D allele (*p* = 0.004). In conclusion, the presence of ACE polymorphism II genotype and I allele may contribute to higher risk of PV and ET; on the other hand, DD genotype and D allele may be associated with reduced likelihood of PV and ET. None of them was significantly associated with thrombotic events [163]. 

Two Toll-like receptor 4 (*TLR4*) SNPs (*TLR4*-D299G and *TLR4*-T399I) were being investigated by Speletas et al. [164], who found no significant correlation between either of the SNPs and thrombosis risk (*p* = 0.43 and *p* = 0.99, respectively). *TLR4*-D299G polymorphism seems to exhibit a protective role in the thrombotic effect, but more studies are needed to confirm this finding. Taken together, in view of the association between gene polymorphism and thrombotic complications in patients with MPN, *GPIIIa*, *XRCC1*, *FBG*, *F5*, *F7*, *MMP9*, *HPA5*, *MTHFR*, and *SDF-1* polymorphisms are related to vascular events, but not for *F2*, *FAS*, *FASL*, *TERT*, *ACE*, and *TLR4*. Thus, it can be speculated that some of the polymorphisms may play a role in the thrombotic risk in patients with MPN; however, larger studies targeting these polymorphisms should be conducted for more clarity.

## 5. Epigenetic Changes in Thrombotic MPN

Other than the acquisition of genetic mutations that give rise to the initiation and development of MPN, the occurrence of the disease may be a result of epigenetic modifications that alter the gene expression by remodelling chromatin. There are two main mechanisms of chromatin remodelling, DNA methylation with the covalent transfer of a methyl group to cytosine-phosphate guanine (CpG) site [165], and post-translational modifications of histones by acetylation, ubiquitination, methylation, phosphorylation, and ADP-ribosylation of glycosylation [166,167]. Another less well-known epigenetic changes will be transcriptional or post-transcriptional regulation of gene expression by non-coding RNAs (ncRNAs), examples for epigenetic related ncRNAs are microRNAs, short interfering RNAs (siRNAs), piwi-interacting RNAs (piRNAs), and long non-coding RNAs (lncRNAs) [168]. Non-coding RNAs can either play their roles through repressing protein translation or causing mRNA degradation [169]. In MPN, there are two categories of epigenetic dysregulations. The first is the presence of gene mutations in genes that encode proteins which regulate the chromatin structure, such as *ASXL1* [132], *TET2* [123,170,171], *IDH1/2* [172,173,174], *EZH2* [175], *IKZF1* [176], *JAK2* V617F [177,178], and *PRMT5* [179]. The second category involves the methylation status of promoter sites of genes that coordinate cell growth, differentiation, and survival [180], such as *CALCA* [181], *ABL1* [182], *SFRP2* [183], *WIF-1* [184], *SOCS1* [185,186], *SOCS3* [187,188], *PRV1* [189], *CXCR4* [190], and *RARβ2* [186,191]. Despite the discovery of epigenetic alterations in MPN, up to now, only the suppressor of cytokine signalling (SOCS) family was elucidated for their relationship with thrombosis in MPN patients.

The methylation status of an important negative regulator family, namely SOCS family including SOCS1, SOCS2, and SOCS3, was studied. Only *SOCS1* and *SOCS2* genes were found to be hypermethylated in five MPN patients, and none of them exhibited thrombotic event. Hence, the hypermethylation status of *SOCS* genes was considered not associated with the incidence of vascular complications in MPN patients [192]. Further study of DNA methylation pattern of epigenetic modifiers, mutations present in them, along with their association with thrombotic events will be needed to comprehensive the effect of epigenetic on the risk of thrombosis in MPN patients.

## 6. Risk Assessment of Thrombosis in MPN According to Different Gene Mutations

Most of the thrombotic events occur around the time of MPN diagnosis which are the first symptoms observed and is considered in the disease diagnosis process [54,193]. Any thrombotic events occurred within four years before the diagnosis of MPN, and before the usage of anti-thrombotic and/or cytoreductive treatment are defined as prior thrombosis (PrTh) [194]. PrTh is very likely to be influenced by pro-thrombotic factors that may still present at MPN diagnosis stage. In order to find out the potential factors with pro-thrombotic effects and identify their prognostic value for the development of vascular problems in MPN, the biological and clinical characteristics at the time of MPN diagnosis are investigated. The PrTh rate is significantly related to male (*p* < 0.009), older age (*p* < 0.001), presence of cardiovascular risk factors (*p* < 0.001), high WBC count (>10 × 109/L), high haematocrit level (>45%), low platelet count (≤700 × 109/L, *p* < 0.001) [195].

The most important risk factors described in MPN are advanced age (≥60 years), and with a history of thrombosis, patients are stratified into low-risk and high-risk based on these two factors [196]. Recently, *JAK2* mutations and cardiovascular risk factors are listed as the new risk factors for arterial thrombosis due to their prognostic value [197]. For the cardiovascular risk factors that are studied, diabetes increases the risk of arterial thrombosis in PV, hypertension in ET, hyperlipidemia in both ET and PMF [54]. In addition, a strong association was also found between increased thrombosis risk and WBC count >8.4 × 109/L [47].

PrTh is more frequently observed in *JAK2* V617F mutated patients compared to the *JAK2* wild type (*p* = 0.002), and *CALR* mutated patients [195]. Different *JAK2* allele burdens were suggested to predict thrombosis in MPN patients. For arterial thrombosis, the cut-off values of allele burden value for PV, ET, and PMF are >25.7%, >25.0% [198], >34.8% [54], respectively. As for venous thrombosis, the cut-off values for *JAK2* allele burden are >90.4% [198] and >56.7% for PV and PMF [54]. Another driver gene *MPL* mutation causes continuous activation of *MPL* and is associated with platelet hyperactivity and increases the risk for micro-vessel disturbances [141]. Aside from *JAK2* and *MPL*, *CALR* is the other gene with a high rate of mutation in MPN, especially in ET and PMF. With an adverse effect from *JAK2* and *MPL*, the presence of *CALR* mutations is usually accompanied by a lower risk of thrombosis, the mutations present more frequently in younger patients and often distributed in low- and intermediate-risk groups [141].

Aside from the *JAK2*, *CALR* and *MPL* genes that hold the prognostic significance in MPN, other non-driver mutations, such as *ASXL1*, *TET2*, *SRSF2*, *IDH2*, *SH2B3*, *SF3B1*, *U2AF1*, *TP53*, and *EZH2*, were found to not carry any prognostic significance [138]. In routine clinical practice, *JAK2*, *CALR*, and *MPL* are the most common genes used for thrombotic risk assessment [32,197]. We suggest that more studies can be done for the genes that are found to be related to thrombosis in MPN from this review, and apply those genes in the evaluation of MPN thrombotic risk to achieve a more comprehensive prognosis for the occurrence of vascular events in PV, ET, and PMF patients.

## 7. Pathogenesis in Thrombotic MPN

The pathogenesis of thrombosis in MPN is complicated and caused by multiple factors. Thrombosis may occur due to platelet activation, high production of tissue factor, formation of platelet-neutrophil aggregates, increased RBC mass [199], imbalance in the coagulation cascade and endothelial cell dysfunction [200]. The details for the pathogenesis of thrombosis are illustrated in Figure 1B.

Hyperactive JAK2-dependent signalling promotes cell-intrinsic defects which promote prothrombotic phenotype, and finally hemorrhage in MPN. Mechanism of thrombosis was depicted from the elevated level of circulating platelets, neutrophils, and erythrocytes due to the disturbed pathway of hematopoietic stem cell function. Their cellular interactions create a hyper-adhesive and prothrombotic milieu predisposing to venous, arterial, or microvascular thrombosis in MPN. The activated platelets can mediate extrinsic coagulation pathway via endothelial P-selectin dependent mechanism, leading to enhance interaction with leukocytes (predominant in neutrophils). Platelet binds to leukocytes through the activated endothelial cell via platelet surface receptors, GPIbα and PSGL-1, and mediate the formation of platelet-leukocyte aggregates. Increased expression of P-selectin, CD40 ligand, and tissue factor (TF) signal a hyperactivated state of platelets [201]. Activated neutrophils ejects their cellular components to form neutrophil extracellular traps (NETs) to induce a hypercoagulable state [9]. Phosphorylation of erythroid Lutheran glycoprotein, Lu, resulted in increased RBC binding to the endothelium. All of these faulty prothrombotic environments enhanced tissue factor, such as activated factor VII (FVIIa) activity, which eventually activate aberrant coagulation cascade. The disrupted endothelial cells due to mutation leading to an increased in marrow and splenic microvessel density and neo-angiogenesis, stimulating production of procoagulant and anti-fibrinolytic proteins, such as Willebrand factor (VWF) deficiency, and plasminogen activator inhibitor 1 (PAI-1) [202]. Activated blood cells and endothelial cells stimulate production of inflammatory cytokines; reactive oxygen species (ROS), hypoxia-inducible factors (HIFα), and tumour necrosis factor α (TNF-α) give rise to chronic proinflammatory state of MPN [203].

## 8. Currently Available Drugs and Therapies for Thrombotic MPN

### 8.1. Hydroxyurea

Hydroxyurea (HU) or hydroxycarbamide acts as the first line cytoreductive therapy in high-risk PV and ET patients [105,204,205]. This drug contains myelosuppressive activity, anti-thrombotic effect [204], anti-metabolite effect [206], and did not enhance the risk of leukaemia transformation in patients [207]. Moreover, this cytoreduction therapy helps to prevent clot formation in high-risk PV [208,209], particularly in case of the thrombosis occurs outside of the splanchnic venous system [210]. Similar effect was found in high-risk ET patients, where the risk for them to develop additional thrombotic episodes was reduced [211]. Besides, a lower dose of HU substantially reduces white cell counts, platelet counts, and haemoglobin concentration in *JAK2* V617F positive patients [212]. However, a significant number of discontinuations of cytoreduction therapies were observed because of the presence of cytopenias or development of intolerance in patients [210]. Alternative therapies and non-leukemogenic drugs such as anagrelide or interferon-alpha (IFN-α) should be considered for patients who develop severe HU-related toxicities [213]. The current baseline level of extreme thrombocytosis in *CALR*-mutated ET (>60 years old) as suggested by European LeukemiaNet (ELN) was >1500 × 10⁹ platelets/L to proceed with HU, whereas for adolescences (<60 years old), pegylated- IFN-α was preferred [214]. This study showed that HU therapy caused a significant decrease in *JAK2 V617F* allele load after 36 months in PV and ET patients [215]. However, PV and ET patients who presented an additional non-driver mutation, such as *TP53*, *ASXL1*, *RUNX1*, *SRSF2* and *IDH2/2*, have persistent increase in *JAK2 V617F* allele burden while receiving HU treatment. This genomic instability likely contributed to myelofibrotic transformation and acute myeloid leukemia [216]. 

### 8.2. Interferon-Alpha

Same as HU, IFN-α is commonly used for cytoreduction as well [205]. IFN-α contains an anti-proliferative effect which helps to induce cytogenetic remission and reduce *JAK2* V617F allele burden [217]. IFN-α also inhibits bone marrow fibroblast progenitor cells and confronts the activity of transforming growth factor, platelet-derived growth factor and other cytokines [218]. Despite that, therapy discontinuation is noted in IFN-α treated patients, since it shows a certain degree of association with side effects [217]. Most patients experience fever and flu-like symptoms after taking the drug and require paracetamol to relieve the symptoms. The long-term usage of IFN-α is limited in which interferon toxicity also causes weakness, weight and hair loss, myalgia, and severe depression. The problem can be solved by using pegylated forms of IFN-α [218]. In PV patients who proved to be intolerant to IFN-α, HU can be substituted for the drugs or vice versa [219]. Recombinant IFN-α and ruxolitinib are suggested in cases with HU intolerance as second-line therapies for PV patients [220]. In younger PV patients (<60 years old) with no history of thrombotic events, at least one of these criteria must be fulfilled before proceeding with the cytoreductive treatment, including unresponsive to phlebotomy, persistent and progressive leukocytosis (>15 × 10^9^ WBC/L), symptomatic progressive splenomegaly, poor haematocrit control, and patient with high cardiovascular risk and disease burden [221]. 

### 8.3. Anagrelide

Anagrelide contains anti-platelet activity without the leukemogenic effect. [211]. Anagrelide has been approved by the Food and Drug Administration (FDA) and acts as a first-line agent to control the thrombocytosis in MPN [213]. For JAK2 V617F negative ET patients, anagrelide provides a potent protection from thrombosis and is suitable to replace HU as a second-line therapy if the patients cannot tolerate HU [211,222]. On the contrary, therapy with anagrelide showed an increased incidence of arterial thrombosis, myelofibrotic transformation, major bleeding events and a decreased rate of venous thrombosis as compared to HU. Treatment with anagrelide sometimes is accompanied by a progression to anaemia and the occurrence of bone marrow fibrosis [212].

### 8.4. Ruxolitinib

Patients without the classical polycythaemia and thrombocythaemia symptoms, since they start at a lower baseline compared to other MPN patients, tend to have lower blood cell counts if treated with cytoreduction drugs like HU and IFN-α. In this patient group, JAK inhibitor ruxolitinib (RUX) would be a better choice [223]. RUX is potential to reduce thrombosis [224], increase cell apoptosis [225], and has been approved by the European Medicines Agency and the FDA as a second-line therapy for PV patients who are intolerant to HU and treatment for intermediate and high-risk PMF [226]. Still, its usage may be limited since ruxolitinib is somehow associated with cytopenias. However, given the high haemorrhage risk evident in MPN patients, especially those with abormal JAK/STAT signalling due to gene mutations, it is important to study more different ways that can direct inhibit JAK/STAT pathway with minimal adverse effect, then can subsequently reduce thrombosis in patients [210]. The current recommendation of the RUX usage is in patients with myelofibrosis-associated splenomegaly (intermediate-2 or high-risk disease) or in case of intermediate-1 disease, as the first-line approach in highly symptomatic splenomegaly [220]. The use of RUX is largely associated with reduced thrombotic risk in PV and ET [227]. 

### 8.5. Momelotinib

From the perspective of symptoms resolution, momelotinib (MMB) is more inferior when compared to RUX. MMB helps to decrease the need for a transfusion. Anaemia and thrombocytopenia may arise when MMB is used for treatment; however, most of the patients show improvement in their life quality [228].

### 8.6. Fedratinib

Similar to RUX and MMB, fedratinib (FED) is a JAK2 inhibitor kinase. FED improves the spleen response in FED treated MPN patients [229] and the obvious result was seen in PMF patients who are intolerant or resistant to RUX. However, the study with FED was terminated when 7% of the patients died due to encephalopathy [230]. Recently, FDA decided to lift the clinical hold since a few FED trials demonstrated that the prevalence of encephalopathy is not more than 1% [231].

### 8.7. Pacritinib

PAC inhibits kinase proteins such as *JAK2* and FLT3 and induces spleen reduction with only a few haematologic toxicities [232]. PAC was suggested to be given to PMF patients and pancytopenia, particularly thrombocytopenia [233].

### 8.8. Busulfan

Busulfan is grouped as second-line cytoreductive therapy. Low dose busulfan can control the haematologic parameters in PV and ET patients, and it is recommended to be used in elderly patients [234]. However, this reagent is only reserved for specific situations, it may induce a higher chance of leukaemic transformation and increase the chance of developing secondary malignancies [235,236]. Efficacy of busulfan was assessed in 36 patients with advanced PV or ET refractory or intolerant to HU, which showed lower probability of thrombosis, 11% and 83% achieved complete hematological response [237]. However, busulfan give little disease-modifying activity because it is not targeting the mediator in the JAK-STAT pathway. The symptom re-appeared when the therapy discontinued. Instead of busulfan, RUX therapy has been given credit on reducing the incidence of thrombosis compared to contemporary therapies like HU or busulfan [227]. 

### 8.9. Aspirin

Aspirin, as an antiplatelet agent, has been proved to ameliorate the risk of thrombosis in PV and ET effectively. Low dose aspirin (100 mg daily) in low-risk PV shows a reduction in VTE and major bleeding. In a higher-risk PV group, a combination of aspirin and clopidogrel works better compared to aspirin alone. However, caution must be taken as the combination treatment may increase severe bleeding in patients [205]. *CALR* mutated ET patients tend to have a lower risk of thrombosis [141]. The use of antiplatelet agents in ET with *CALR* mutations may cause undesirable effects and results in harm than bringing benefit to the patients [238]. The current recommendations by European LeukemiaNet (ELN) were the use of aspirin at low dose (75–100 mg/day) only in classical low-risk group of *CALR*-mutated ET patients [214].

### 8.10. Anticoagulants

Anticoagulants display high efficacy in preventing venous VTE among the general population [205]. Anticoagulants are sometimes used together with antiplatelet agents to lower rates of thrombosis, but this combination therapy was observed to result in high bleeding risk in a specific population. Thus, care must be taken for the usage of this strategy [239]. Over the past few years, traditional anticoagulant vitamin K antagonist was gradually replaced by direct oral anticoagulants (DOACs) in the management of thrombotic complications due to their greater effectiveness in blood malignancy [240]. Experts suggested the use of DOACs, such as low-molecular-weight heparin or fondaparinux, as initial therapy prior to the use of conventional vitamin K antagonist to reduce risk of VTE recurrent [241], and this is a valid therapeutic over warfarin for prolonged thromboprophylaxis [242].

### 8.11. Phlebotomy

Over 20 years ago, the “Polycythaemia Vera Study Group” observed that PV patients undergoing phlebotomy displays a lower incidence of acute leukaemia and other malignancies, thus tend to have better overall median survival [243], however, an increased risk of thrombosis was detected along with the therapy [244]. In clinical practice, an amount of 250–500 cm^3^ blood should be withdrawn daily or every other day until the haematocrit level reaches between 40 and 45%. A smaller amount of 200–300 cm^3^ blood was suggested to be withdrawn twice a week for the elderly or those with cardiovascular diseases [243,245]. The strong evidenced-based recommendations by the Italian Society of Hematology (SIE) for phlebotomy in PV were; a hematocrit <45% or a lower target hematocrit (40–42%) in patients with persistent hyper-viscosity. Platelet-lowering drugs or increased doses of the drug should not be given to a patient with post-phlebotomy thrombocytosis due to poor association with increased risk of vascular event, and to perform RBC apheresis instead of phlebotomy in patient with a severe vascular complications [246]. 

### 8.12. Radiophosphorus and Chlorambucil

Cytoreduction therapy with radiophosphorus or chlorambucil alleviates thrombotic events in PV patients. Nevertheless, the drugs are accompanied by increase leukemogenicity, which eventually leads to shorter survival [243].

### 8.13. Allogenic Stem-Cell Transplantation

This therapy is recommended for MF patients with high or intermediate-2 risk score group. An exceptional cases of MF patients with intermediate-1 risk score was also recommended who reached strict criteria; refractory, transfusion-dependent anaemia, more than 2% blasts in peripheral blood, has adverse cytogenetics abnormalities, or high-risk mutations [220].

## 9. Conclusions

Although many gene mutations were detected in MPN, the causative roles of these mutations in thrombosis are not completely understood. The investigation of these major gene drivers such as *JAK2*, *ASXL1*, *MPL*, *CALR*, and *TET2*, and some SNPs are in progress. In this review, we concluded that a strong correlation between *JAK2* V617F and *ASXL1* gene mutations and thrombotic events are consistent. *MPL* likely have no relationship to thrombosis. As for *JAK2* exon 12 polymorphisms, *CALR* and *TET2* gene polymorphisms, their association with the risk of thrombotic complications remained unclear. Gene polymorphisms in *GPIIIa*, *XRCC1*, *FBG*, *F7*, *MMP9*, *MTHFR*, and *SDF-1* may also contribute to thrombotic complications in patients with MPN, but not polymorphisms in *HPA5*, *FAS*, *FASL*, *TERT*, *ACE*, and *TLR4* genes that have little to no association with thrombotic events in MPN. On the other hand, *HPA5* and *TLR4*-D229G exhibit a possible protective role to thrombosis in patients with MPN. In short, more studies are needed, and perhaps a large-scale study with large sample sizes is necessary to delineate the effects of these mutations on the pathogenesis of thrombosis in MPN and the gene-to-gene interactions that may share the common diseases signalling pathways that lead to the development of thrombosis in various sites seen in MPN patients.

## Figures and Tables

**Table 1:** Bone marrow trephine biopsy of classical BCR-ABL-negative MPN under 20× and 40×. (**A**,**B**) Polycythaemia vera. (**C**,**D**) Essential thrombocythaemia. (**E**,**F**) Primary myelofibrosis.

Type of MPN	Polycythaemia Vera (PV)	Essential Thrombocythaemia (ET)	Primary Myelofibrosis (PMF)
Bone marrow trephine biopsy under 20×	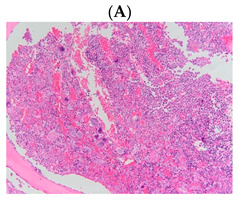	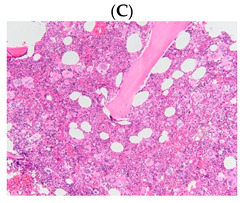	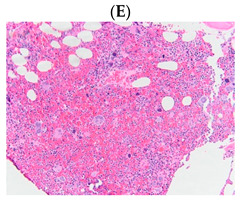
Bone marrow trephine biopsy under 40×	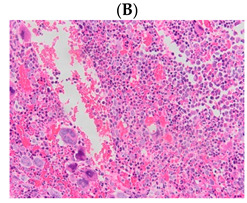	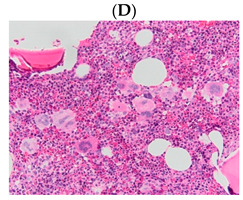	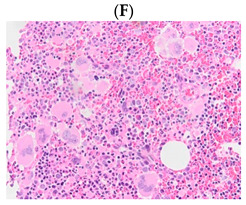
Description	Hypercellularity, panmyelosis with a notable predominance of megakaryocytic and erythroid lineages, and megakaryocytes increase in size and with frequent hyperlobated forms, minimal or absence of reticulin fibrosis.	Normal or mildly hypercellularity, a normal proliferation of erythroid and granulocytic lineages, enlarged megakaryocytes (mature with hyperlobulated nuclei), delicate reticulin fibres.	Hypercellularity with clustered and dysplastic large size megakaryocytes, presence of hyperlobulated and hyperchromatic megakaryocytes, often naked megakaryocytes, increase reticulin around clustered megakaryocytes.

## Data Availability

Data are contained within the article.

## Supplementary Materials

The following supporting information can be downloaded at: https://www.mdpi.com/article/10.3390/diagnostics13010163/s1. Table S1: Thrombotic MPN patients with different gene mutations and gene; Table S2: Summary of *JAK2*, *CALR*, *MPL*, *TET2*, *ASXL1* and gene mutation and polymorphisms in thrombotic MPN patients.
