# Supplementary material for: Molecular Genetics of Thrombotic Myeloproliferative Neoplasms: Implications in Precision Oncology
Source: Diagnostics (Basel). 2023 Jan 3;13(1):163. doi: 10.3390/diagnostics13010163 (PMC9818412; doi:10.3390/diagnostics13010163)
Supplement: Supplementary file 1 [file diagnostics-13-00163-s001.zip › diagnostics-1905673-supplementary.pdf]

Table S1. Thrombotic MPN patients with different gene mutations and gene polymorphisms.

Type of patients	Total number of patients	Number of thrombotic patients (%)	References
**Tyrosine kinase**			
***JAK*2 V617F**			
PV	40	14 (35.0)	[1]
	16	3 (18.8)	[2]
	35	8 (22.9)	[3]
	215	28 (13.0)	[4]
	53	18 (34.0)	[5]
	35	15 (42.9)	[6]
	69	14 (20.3)	[7]
	31	11 (35.5)	[8]
	323	110 (34.1)	[9]
ET	30	14 (46.7)	[1]
	31	13 (41.9)	[2]
	77	35 (45.5)	[3]
	153	27 (17.6)	[4]
	55	17 (30.9)	[5]
	60	28 (46.7)	[10]
	22	7 (31.8)	[6]
	466	33 (7.1)	[11]
	369	111 (30.1)	[12]
	21	3 (14.3)	[13]
	35	4 (11.4)	[14]
	567	48 (8.5)	[15]
	179	39 (21.8)	[7]
	35	10 (28.6)	[8]
	382	138 (36.1)	[9]
PMF	4	0 (0.0)	[2]
	54	9 (16.7)	[4]
	7	0 (0.0)	[5]
	16	5 (31.3)	[6]
	6	0 (0.0)	[16]
	6	0 (0.0)	[8]
MPN	32	20 (62.5)	[17]
	59	18 (30.5)	[18]
	273	84 (30.8)	[19]
	126	39 (31.0)	[20]
	200	169 (84.5)	[21]
	119	45 (37.8)	[22]
	44	23 (52.3)	[23]
	123	58 (47.2)	[24]
	209	84 (40.2)	[25]
	44	38 (86.4)	[26]
PV or ET	36	9 (25.0)	[27]
	74	34 (45.9)	[28]

***JAK2* exon12**			
PV	4	1 (25.0)	[7]
**Receptors**			
MPL			
ET	25	10 (40.0)	[12]
PMF	2	0 (0.0)	[29]
ET or PMF	32	11 (34.4)	[30]
CALR			
ET	85<br>176<br>89<br>23<br>10<br>208<br>47	6 (7.1)<br>5 (2.8)<br>12 (13.5)<br>5 (21.7)<br>0 (0.0)<br>7 (3.4)<br>3 (6.4)	[4]<br>[11]<br>[12]<br>[13]<br>[16]<br>[15]<br>[7]
PMF	21<br>3	1 (4.8)<br>0 (0.0)	[4]<br>[16]
MPN	91	9 (9.9)	[31]
FAS AA			
MPN	32	10 (31.3)	[32]
FAS AG + GG			
MPN	69	16 (23.2)	[32]
TLR4 D299G			
MPN	18	4 (22.2)	[22]
TLR4 T399I			
MPN	18	6 (33.3)	[22]
**DNA methylation**			
TET2			
PV	4<br>14<br>20	0 (0.0)<br>2 (14.3)<br>13 (65.0)	[33]<br>[34]<br>[35]
ET	6<br>3<br>1	3 (50.0)<br>3 (100.0)<br>1 (100.0)	[33]<br>[34]<br>[36]
PMF	2<br>0<br>2	1 (50.0)<br>10 (0.0)<br>2 (100.0)	[33]<br>[34]<br>[36]
**Histone methylation**			
ASXL1			
ET	14	6 (42.9)	[13]
***DNMT3A* + *TET2* + *ASXL1* (DTA)**			
PV	29	19 (65.5)	[35]
**Thrombophilic genetic factors**			
F5			
MPN	11<br>20	2 (18.2)<br>5 (25.0)	[22]<br>[37]

PV or ET	14	5 (35.7)	[38]
F2			
MPN	15<br>7	6 (40.0)<br>1 (14.3)	[22]<br>[37]
***GPIIIa* PI^{A1}**			
PV	32	6 (18.8)	[39]
***GPIIIa* PI^{A2}**			
PV	11	6 (54.5)	[39]
MTHFR			
MPN	96	32 (33.3)	[22]
**DNA repair**			
XRCC1			
MPN	78	22 (28.2)	[40]
**Chemokine**			
***SDF-1* AA**			
PV or ET	10	7 (70.0)	[41]
***SDF-1* AG + GG**			
PV or ET	63	21 (33.3)	[41]

Abbreviations: *ASXL1*: *Additional Sex Combs Like 1, CALR*: *Calrecticulin,* ET: Essential thrombocythaemia, *F2*: *Factor II, F5*: *Factor 5, GPIIIa PI^{A1}*: *Glycoprotein IIIa PI^{A1}* allele, *GPIIIa PI^{A2}*: *Glycoprotein IIIa PI^{A2}* allele, *JAK2*: *Janus kinase 2,* PMF: Primary myelofibrosis, *MPL*: *Myeloproliferative leukaemia virus oncogene,* MPN: Myeloproliferative neoplasms, *MTHFR*: *Methylenetetrahydrofolate reductase,* PV: Polycythaemia vera, *SDF-1*: *Stromal cell-derived factor-1, TET2*: *Ten-eleven translocation 2, TLR4*: *Toll-like receptor 4, XRCC1*: *X-ray repair cross complementing 1.*

Table S2. Summary of *JAK2*, *CALR*, *MPL*, *TET2*, *ASXL1* and gene mutation and polymorphisms in thrombotic MPN patients.

**MUTATIONS**			
**Genes**	**Descriptions**	***P* value**	**References**
JAK2 V617F	*JAK2* V617F is associated to thrombosis in PV and ET.	0.02	[1]
	Increase the thrombotic risk in MPN.	0.04	[17]
	Thrombosis is more common in MPN patients with the mutation. Allele burden increases progressively with age.	0.006	[42]
	Higher incidence of thrombosis in MPN patients with the mutation.	0.047	[2]
	JAK2 mutation and additional thrombophilic markers predispose MPN patients with thrombocythaemia to thrombosis.	0.017	[43]
	JAK2 is an independent risk factor for thrombosis.	-	[44]
	ET and PMF with *JAK2* mutation are more susceptible to thrombosis.	<0.01	[19]
	JAK2 mutated ET patients showed more frequent thrombotic events.	0.004	[45]
	No significant difference was found between BCS and PVT and *JAK2* V617F.	0.989	[46]
	Higher prevalence of *JAK2* V617F mutation in non-cirrhotic and non-malignant PVT patients.	<0.001	[47]
	Weak association with thrombosis, particularly VTE present in sites other than the splanchnic region.	-	[48]
	Low incidence of *JAK2* V617F in SVT.	-	[49]
	No significant different between ET patients with or without mutation.	0.57	[50]
	Patients with 12.6-30% and 78-100% *JAK2* V617F allele burden have higher rate of arterial thrombosis. Allele burden can predict the site of thrombosis but had no effect on thrombosis timing.	0.001	[28]
	Patients with *JAK2* V617F allele burden >20% had 7.4-fold increased VTE risk.	0.004	[20]
	Risk of VTE increased as JAK2 V617F allele burden is higher.	<0.05	
	The allele burden of *JAK2* V617F correlate to thrombotic events in ET.	0.01	[51]
	Related to the thrombotic events before or during diagnosis.	<0.03	[10]
	More frequent thrombosis in those with homozygous *JAK2* V617F.	0.003	[21]
	PV patients with >75% *JAK2* V617F allele burden displayed higher thrombotic risk compared to <25%.	0.003	[52]
	Increase arterial thrombosis in *JAK2* mutated ET, but no association between the allele burden and thrombosis.	0.001	[53]
	Equal number of thrombosis was observed in *JAK2*	-	[5]

	positive and negative groups.		
	Thrombotic risk was not affected by *JAK2* mutation.	0.22	[18]
	Occurred more often in patients with *JAK2* V67F mutation but us statistically insignificant.	0.18	[27]
JAK2 exon 12	Similar thrombotic risk as in *JAK2* V617F mutated PV patients.	0.4	[54]
CALR	Less frequent venous thrombosis in ET with *CALR* mutation compared to *JAK2* mutation.	0.03	[4]
	Lower risk to develop thrombosis in ET and PV with *CALR* mutation compared to *JAK2* mutation.	0.001	[11]
	ET has lower risk to develop thrombotic events.	0.01	[12]
MPL	No obvious differences in the frequency of thrombosis compared to other mutations. Exhibit micro vascular symptoms.	>0.05	[12]
	No correlation was established between MPL mutations and thrombosis.	>0.05	[30]
	No vascular complications observed in PMF patients with mutation.	-	[29]
TET2	Not related to the occurrence of thrombotic events in all subtypes of MPN.	0.422	[33]
	Significantly accompanied by the development of thrombosis in PV	0.03	[35]
ASXL1	Exhibit higher probability to develop thrombosis. A risk factor for vascular complications in ET.	0.021	[13]
	Leukocytosis, shorter overall survival in PMF.	<0.001	[55]
	**POLYMORPHISMS**		
GPIIIa PIA2 allele	Higher chance to develop arterial thrombosis in PV patients with homozygous or heterozygous PIA2 polymorphism.	<0.05	[39]
HPA-1a/1b variant	No correlation was found with thrombotic events.	-	[56]
GPIa/IIa c.807C>T	Higher prevalence of TT genotype in MPN patients with arterial thrombosis.	0.049	[57]
XRCC1 Gln399Arg	Associated with thrombotic and bleeding complications in MPN patients.	0.386	[40]
	CT genotype was detected more higher in MPN patients with thrombotic events compared to homozygous and wild type.	0.02	[57]
F2 (Prothrombin's G20210A)	Not related to thrombosis in PV and ET.	-	
	No relationship observed between *F2* and thrombosis in MPN.	>0.05	[37]
F5 (Leiden	Not related to thrombosis in PV and ET.	-	[57]

mutation)	No relationship observed between *F5* and thrombosis in MPN.	>0.05	[37]
	Higher rate of detection of venous thrombosis before and at time of diagnosis and recurrence of venous thrombosis, but not arterial thrombosis.	0.03	[38]
	Increase the risk of thrombosis in MPN patients.	0.03	[58]
	Strong association with increased thrombotic risk.	0.019	[48]
F7	Independent risk factor in thrombotic development of overall MPN.	0.0007	[59]
F7 (c.-323P0/10)	More frequently present in thrombotic MPN patients.	0.04	[57]
F12 -46C/T	Not correlate to thrombotic complication in PV and ET.	-	[56]
MMP9	Might be a contributing factor for the occurrence of vascular events in MPN patients, especially in ET group.	0.002	[60]
HPA5	*HPA5 b* allele has a protective role for vascular events in MPN patients.	0.03	[61]
MTHFR-C667T	Could have a role as a prothrombotic factor and causing thrombosis in MPN patients.	<0.05	[62]
SDF-1	Homozygous AA PV and ET patients tend to have thrombosis. AA genotype can be as a predictor for thrombotic events in PV and ET patients.	0.03	[41]
FAS 670A>G	No association to venous thrombosis.	0.41	[32]
FASL 843C>T	No statistically significant correlation between the polymorphism and venous thrombosis.	>0.05	
TERT	Not related to thrombosis.	-	[63]
ACE I and *D* allele	Both showed no associated to thrombotic events.	>0.05	[64]
TLR4-D299G	Irrelevant to thrombotic risk. Seems to exhibit a protective role in thrombotic effect.	0.43	[22]
TLR4-T399I	Irrelevant to thrombotic risk.	0.99	

Abbreviations: *ASXL1*: *Additional Sex Combs Like 1*, *CALR*: *Calrecticulin*, ET: Essential thrombocythaemia, *F2*: *Factor II*, *F5*: *Factor 5*, *GPIIIa* PI^{A1}: *Glycoprotein IIIa* PI^{A1} allele, *GPIIIa* PI^{A2}: *Glycoprotein IIIa* PI^{A2} allele, *JAK2*: *Janus kinase 2*, PMF: Primary myelofibrosis, *MPL*: *Myeloproliferative leukaemia virus oncogene*, MPN: Myeloproliferative neoplasms, *MTHFR*: *Methylenetetrahydrofolate reductase*, PV: Polycythaemia vera, *SDF-1*: *Stromal cell-derived factor-1*, *TET2*: *Ten-eleven translocation 2*, *TLR4*: *Toll-like receptor 4*, *XRCC1*: *X-ray repair cross complementing 1*.

References

1. Basquiera, A.L.; Soria, N.W.; Ryser, R.; Salguero, M.; Moiraghi, B.; Sackmann, F.; Sturich, A.G.; Borello, A.; Berretta, A.; Bonafé, M. Clinical significance of V617F mutation of the JAK2 gene in patients with chronic myeloproliferative disorders. *Hematology* **2009**, *14*, 323-330.
2. Takata, Y.; Seki, R.; Kanajii, T.; Nohara, M.; Koteda, S.; Kawaguchi, K.; Nomura, K.; Nakamura, T.; Morishige, S.; Oku, E. Association between Thromboembolic Events and the JAK2 V617F Mutation in Myeloproliferative Neoplasms Cancer. *Kurume Med. J.* **2014**, *60*, 89-97.
3. Speletas, M.; Katodritou, E.; Daiou, C.; Mandala, E.; Papadakis, E.; Kioumi, A.; Ritis, K.; Korantzis, I. Correlations of JAK2–V617F mutation with clinical and laboratory findings in patients with myeloproliferative disorders. *Leuk. Res.* **2007**, *31*, 1053-1062.

4. Andrikovics, H.; Krahling, T.; Balassa, K.; Halm, G.; Bors, A.; Koszarska, M.; Batai, A.; Dolgos, J.; Csomor, J.; Egyed, M. Distinct clinical characteristics of myeloproliferative neoplasms with calreticulin mutations. *Haematologica* **2014**, *99*, 1184-1190.
5. Iványi, J.L.; Marton, É.; Plander, M. Significance of the JAK2V617F mutation in patients with chronic myeloproliferative neoplasia. *Orv. Hetil.* **2011**, *152*, 1795-1803.
6. Mattar, M.M.; Nassef, S.; El Husseiny, N.M.; El Masry, M.R.; Salah, M.; Morad, M.A.; Gawad, A.A. Incidence of Silent Thrombosis in Patients Younger Than 60 Years With Myeloproliferative Neoplasms: Single-Center Egyptian Study. *Clin. Lymphoma Myeloma Leuk.* **2019**, *19*, 425-429.
7. Okabe, M.; Yamaguchi, H.; Usuki, K.; Kobayashi, Y.; Kawata, E.; Kuroda, J.; Kimura, S.; Tajika, K.; Gomi, S.; Arima, N., et al. Clinical features of Japanese polycythemia vera and essential thrombocythemia patients harboring CALR, JAK2V617F, JAK2Ex12del, and MPLW515L/K mutations. *Leuk. Res.* **2016**, *40*, 68-76.
8. Marton, S.; Ivanyi, L.; Kereskai, L.; Pajor, L. Incidence of jak2 v617f tyrosine kinase mutation and its correlation with thrombosis and bleeding complications in patients with chronic myeloproliferative disorders. *Haematologica* **2008**, *93*, 524-524.
9. Vannucchi, A.M.; Antonioli, E.; Guglielmelli, P.; Rambaldi, A.; Barosi, G.; Marchioli, R.; Marfisi, R.M.; Finazzi, G.; Guerini, V.; Fabris, F. Clinical profile of homozygous JAK2 617V> F mutation in patients with polycythemia vera or essential thrombocythemia. *Blood* **2007**, *110*, 840-846.
10. Patriarca, A.; Pompetti, F.; Malizia, R.; Iuliani, O.; Di Marzio, I.; Spadano, A.; Dragani, A. Is the absence of JAK2V617F mutation a risk factor for bleeding in essential thrombocythemia? An analysis of 106 patients. *Blood Transfus.* **2010**, *8*, 21-27.
11. Rumi, E.; Pietra, D.; Ferretti, V.; Klampfl, T.; Harutyunyan, A.S.; Milosevic, J.D.; Them, N.C.; Berg, T.; Elena, C.; Casetti, I.C. JAK2 or CALR mutation status defines subtypes of essential thrombocythemia with substantially different clinical course and outcomes. *Blood* **2014**, *123*, 1544-1551.
12. Rotunno, G.; Mannarelli, C.; Guglielmelli, P.; Pacilli, A.; Pancrazzi, A.; Pieri, L.; Fanelli, T.; Bosi, A.; Vannucchi, A.M. Impact of calreticulin mutations on clinical and hematological phenotype and outcome in essential thrombocythemia. *Blood* **2014**, *123*, 1552-1555.
13. Nie, Y.B.; Sun, M.; He, C.K.; Ju, M.K.; Zhou, F.L.; Wu, S.Y.; Zhou, Y.; Liu, L.; Shen, H.; Huang, T.T. ASXL1 mutations in Chinese patients with essential thrombocythemia. *Exp. Ther. Med.* **2018**, *15*, 4149-4156.
14. Tognon, R.; de Souza Nunes, N.; de Castro, F.A. Apoptosis deregulation in myeloproliferative neoplasms. *Einstein* **2013**, *11*, 540-544.
15. Pietra, D.; Rumi, E.; Ferretti, V.; Di Buduo, C.; Milanesi, C.; Cavalloni, C.; Sant'Antonio, E.; Abbonante, V.; Moccia, F.; Casetti, I. Differential clinical effects of different mutation subtypes in CALR-mutant myeloproliferative neoplasms. *Leukemia* **2016**, *30*, 431-438.
16. Tang, Q.; Zhang, X.; Xia, L.; Jiang, N. Mutation of CALR Gene in Patients with Chronic Myeloproliferative Neoplasm and Its Clinical Significance. *Chin. J. Exp. Trad. Med. Form.* **2017**, *25*, 151-156.
17. Singh, K.; Sazawal, S.; Chhikara, S.; Mahapatra, M.; Saxena, R. Association of JAK2V617F mutation with thrombosis in Indian patients with Philadelphia negative chronic myeloproliferative neoplasms. *Indian J. Pathol. Microbiol.* **2018**, *61*, 371-374.
18. Lieu, C.H.; Wu, H.S.; Hon, Y.C.; Tsai, W.H.; Yang, C.F.; Wang, C.C.; Lin, Y.C.; Shih, C.H.; Hsu, H.C. Prevalence of the JAK2-V617F mutation in Taiwanese patients with chronic myeloproliferative disorders. *Intern. Med. J.* **2008**, *38*, 422-426.
19. Guo, X.; Yang, L.; Yan, K.; Yang, R.; Lin, J. Relationship between JAK2-V617F Gene Mutation in Peripheral Blood Mononuclear Cells and Thrombotic Events in Patients with Myeloproliferative Neoplasms. *J. Mod. Lab. Med.* **2017**, *32*, 143-145.
20. Borowczyk, M.; Wojtaszewska, M.; Lewandowski, K.; Gil, L.; Lewandowska, M.; Lehmann-Kopydłowska, A.; Kroll-Balcerzak, R.; Balcerzak, A.; Iwoła, M.; Michalak, M., et al. The JAK2 V617F mutational status and allele burden may be related with the risk of venous thromboembolic events in patients with Philadelphia-negative myeloproliferative neoplasms. *Thromb. Res.* **2015**, *135*, 272-280.
21. Bang, S.-M.; Lee, J.-S.; Ahn, J.Y.; Lee, J.H.; Hyun, M.S.; Kim, B.S.; Park, M.R.; Chi, H.-S.; Kim, H.Y.; Kim, H.J. Vascular events in Korean patients with myeloproliferative neoplasms and their relationship to JAK2 mutation. *Thromb. Haemostasis* **2009**, *101*, 547-551.
22. Speletas, M.; Liadaki, K.; Kalala, F.; Daiou, C.; Katodritou, E.; Mandala, E.; Korantzis, I.; Ritis, K.; Zintzaras, E.; Germenis, A. TLR4 single nucleotide polymorphisms and thrombosis risk in patients with myeloproliferative disorders. *Thromb. Res.* **2008**, *122*, 27-32.
23. Li, Z.; Fu, H.; Wang, Z.; Yang, S.; Xu, H. Correlative study between the JAK2V617F mutation and thrombosis in patients with myeloproliferative neoplasm. *Genet. Mol. Res.* **2016**, *15*, 1-7.
24. Tevet, M.; Ionescu, R.; Dragan, C.; LUPU, A.R. Influence of the JAK2 V617F mutation and inherited thrombophilia on the thrombotic risk among patients with myeloproliferative disorders. *Maedica* **2015**, *10*, 27-32.
25. Giamouris, V.; Befani, M.; Papadakis, E.; Mandala, E.; Papadoulis, N.; Karamagiolis, S.; Gastari, V.; Verrou, E.; Katodritou, E.; Lafioniatis, S. Influence of common thrombophilia polymorphisms on the thrombosis risk in patients with JAK2-V617F-positive myeloproliferative neoplasms. *Thromb. Res.* **2012**, *130*, 117-118.
26. Kundranda, M.N.; Maiti, B.; Iqbal, N.; Muslimani, A.A.; Chaudhry, A.; Spiro, T.M.; Daw, H. The association of leukocytosis, thrombocytosis and JAK2V617F mutation with thrombotic events in myeloproliferative disorders (MPD's). *Blood* **2008**, *112*, 2803.
27. Hattori, N.; Fukuchi, K.; Nakashima, H.; Maeda, T.; Adachi, D.; Saito, B.; Yanagisawa, K.; Matsuda, I.; Nakamaki, T.; Gomi, K. Megakaryopoiesis and platelet function in polycythemia vera and essential thrombocythemia patients with JAK2 V617F mutation. *Int. J. Hematol.* **2008**, *88*, 181-188.

28. Uyanik, M.S.; Baysal, M.; Pamuk, G.E.; Maden, M.; Akker, M.; Umit, E.G.; Demir, M.; Aydogdu, E. Is JAK2V617F mutation the only factor for thrombosis in Philadelphia-negative chronic myeloproliferative neoplasms? *Indian J. Hematol. Blood Transfus.* **2016**, *32*, 262-267.
29. Akpınar, T.S.; Hançer, V.S.; Nalçacı, M.; Diz-Küçükkaya, R. MPL W515L/K mutations in chronic myeloproliferative neoplasms. *Turk. J. Haematol.* **2013**, *30*, 8-12.
30. Beer, P.A.; Campbell, P.J.; Scott, L.M.; Bench, A.J.; Erber, W.N.; Bareford, D.; Wilkins, B.S.; Reilly, J.T.; Hasselbalch, H.C.; Bowman, R. MPL mutations in myeloproliferative disorders: analysis of the PT-1 cohort. *Blood* **2008**, *112*, 141-149.
31. Mózes, R.; Gángó, A.; Sulák, A.; Vida, L.; Reiniger, L.; Timár, B.; Krenács, T.; Alizadeh, H.; Masszi, T.; Gaál-Weisinger, J., et al. Calreticulin mutation specific CAL2 immunohistochemistry accurately identifies rare calreticulin mutations in myeloproliferative neoplasms. *Pathology* **2019**, *51*, 301-307.
32. Ozdemirkiran, F.G.; Nalbantoglu, S.; Gokgoz, Z.; Payzin, B.K.; Vural, F.; Cagirgan, S.; Berdeli, A. FAS/FASL gene polymorphisms in Turkish patients with chronic myeloproliferative disorders. *Arch. Med. Sci.* **2017**, *13*, 426-432.
33. Ha, J.-S.; Jeon, D.-S.; Kim, J.-R.; Ryoo, N.-H.; Suh, J.-S. Analysis of the Ten-Eleven Translocation 2 (TET2) gene mutation in myeloproliferative neoplasms. *Ann. Clin. Lab. Sci.* **2014**, *44*, 173-179.
34. Tefferi, A.; Pardanani, A.; Lim, K.; Abdel-Wahab, O.; Lasho, T.; Patel, J.; Gangat, N.; Finke, C.; Schwager, S.; Mullally, A. TET2 mutations and their clinical correlates in polycythemia vera, essential thrombocythemia and myelofibrosis. *Leukemia* **2009**, *23*, 905-911.
35. Segura-Díaz, A.; Stuckey, R.; Florido, Y.; González-Martín, J.M.; López-Rodríguez, J.F.; Sánchez-Sosa, S.; González-Pérez, E.; Sáez Perdomo, M.N.; Perera, M.d.M.; de la Iglesia, S. Thrombotic Risk Detection in Patients with Polycythemia Vera: The Predictive Role of DNMT3A/TET2/ASXL1 Mutations. *Cancers* **2020**, *12*, 1-9.
36. Colaizzo, D.; Tiscia, G.; Pisanelli, D.; Bafunno, V.; Amitrano, L.; Grandone, E.; Guardascione, M.; Margaglione, M. New TET2 gene mutations in patients with myeloproliferative neoplasms and splanchnic vein thrombosis. *J. Thromb. Haemost.* **2010**, *8*, 1142-1144.
37. Soyer, N.; Küçükarslan, A.S.; Sahin, F.; Çekdemir, D.; Kosova, B.; Eroglu, Z.; Töbü, M.; Tombuloglu, M.; Çagirgan, S.; Dönmez, A. Factor V G1691A (Leiden) and prothrombin G20210A gene mutation status, and thrombosis in patients with chronic myeloproliferative disorders/Kronik myeloproliferatif hastalik tanili hastalarda Factor V 1691A (Leiden) ve protrombin G20210A gen mutasyonu ve tromboz. *Turk. J. Haematol.* **2011**, *28*, 306.
38. Ruggeri, M.; Gisslinger, H.; Tosetto, A.; Rintelen, C.; Mannhalter, C.; Pabinger, I.; Heis, N.; Castaman, G.; Missiaglia, E.; Lechner, K. Factor V Leiden mutation carriership and venous thromboembolism in polycythemia vera and essential thrombocythemia. *Am. J. Hematol.* **2002**, *71*, 1-6.
39. Afshar-Kharghan, V.; López, J.A.; Gray, L.A.; Padilla, A.; Borthakur, G.; Roberts, S.C.; Pruthi, R.K.; Tefferi, A. Hemostatic gene polymorphisms and the prevalence of thrombotic complications in polycythemia vera and essential thrombocythemia. *Blood Coagul. Fibrinolysis* **2004**, *15*, 21-24.
40. Azevedo, A.P.; Silva, S.N.; Reichert, A.; Lima, F.; Júnior, E.; Rueff, J. Effects of polymorphic DNA genes involved in BER and caspase pathways on the clinical outcome of myeloproliferative neoplasms under treatment with hydroxyurea. *Mol. Med. Rep.* **2018**, *18*, 5243-5255.
41. Gerli, G.; Vanelli, C.; Turri, O.; Erario, M.; Gardellini, A.; Pugliano, M.; Biondi, M.L. SDF1-3′ A gene polymorphism is associated with chronic myeloproliferative disease and thrombotic events. *Clin. Chem.* **2005**, *51*, 2411-2414.
42. Randi, M.L.; Ruzzon, E.; Tezza, F.; Scapin, M.; Duner, E.; Scandellari, R.; Fabris, F. JAK2V617F mutation is common in old patients with polycythemia vera and essential thrombocythemia. *Aging Clin. Exp. Res.* **2011**, *23*, 17-21.
43. Penka, M.; Schwarz, J.; Doubek, M.; Dulicek, P.; Indrak, K.; Brychtova, Y.; Hlusí, A.; Kissova, J.; Mayer, J.; Pavlik, T. JAK2 Mutation and Additional Thrombophilic Markers Predispose to Thrombosis in Myeloproliferative Diseases with Thrombocythemia. *Blood* **2008**, *112*, 5257.
44. Mahjoub, S.; Baccouche, H.; Sahnoun, M.; Kaabi, H.; Manai, Z.; Slama, H.; Ben, N.R. The JAK2 mutation in myeloproliferative disorders: A predictive factor of thrombosis. *Tunis. Med.* **2015**, *93*, 474-477.
45. Kang, M.-G.; Choi, H.-W.; Lee, J.H.; Choi, Y.J.; Choi, H.-J.; Shin, J.-H.; Suh, S.-P.; Szardenings, M.; Kim, H.-R.; Shin, M.-G. Coexistence of JAK2 and CALR mutations and their clinical implications in patients with essential thrombocythemia. *Oncotarget* **2016**, *7*, 57036-57049.
46. Kiladjian, J.-J.; Cervantes, F.; Leebeek, F.W.; Marzac, C.; Cassinat, B.; Chevret, S.; Cazals-Hatem, D.; Plessier, A.; Garcia-Pagan, J.-C.; Murad, S.D. The impact of JAK2 and MPL mutations on diagnosis and prognosis of splanchnic vein thrombosis: a report on 241 cases. *Blood* **2008**, *111*, 4922-4929.
47. Qi, X.; Zhang, C.; Han, G.; Zhang, W.; He, C.; Yin, Z.; Liu, Z.; Bai, W.; Li, R.; Bai, M. Prevalence of the JAK2V617F mutation in Chinese patients with Budd-Chiari syndrome and portal vein thrombosis: a prospective study. *J. Gastroenterol. Hepatol.* **2012**, *27*, 1036-1043.
48. Singh, N.; Sharma, A.; Sazawal, S.; Ahuja, A.; Upadhyay, A.; Mahapatra, M.; Saxena, R. Prevalence of JAK2V617F mutation in deep venous thrombosis patients and its clinical significance as a thrombophilic risk factor: Indian perspective. *Clin. Appl. Thromb. Hemost.* **2015**, *21*, 579-583.
49. Yoo, E.H.; Jang, J.H.; Park, K.J.; Gwak, G.Y.; Kim, H.J.; Kim, S.H.; Kim, D.K. Prevalence of overt myeloproliferative neoplasms and JAK2 V617F mutation in Korean patients with splanchnic vein thrombosis. *Int. J. Lab. Hematol.* **2011**, *33*, 471-476.
50. Wolanskyj, A.P.; Lasho, T.L.; Schwager, S.M.; McClure, R.F.; Wadleigh, M.; Lee, S.J.; Gary Gilliland, D.; Tefferi, A. JAK2V617F mutation in essential thrombocythaemia: clinical associations and long-term prognostic relevance. *Br. J. Haematol.* **2005**, *131*, 208-213.

51. Ha, J.-S.; Kim, Y.-K.; Jung, S.-I.; Jung, H.-R.; Chung, I.-S. Correlations between Janus kinase 2 V617F allele burdens and clinicohematologic parameters in myeloproliferative neoplasms. *Ann. Lab. Med.* **2012**, *32*, 385-391.
52. Vannucchi, A.; Antonioli, E.; Guglielmelli, P.; Longo, G.; Pancrazzi, A.; Ponziani, V.; Bogani, C.; Ferrini, P.R.; Rambaldi, A.; Guerini, V. Prospective identification of high-risk polycythemia vera patients based on JAK2 V617F allele burden. *Leukemia* **2007**, *21*, 1952-1959.
53. Larsen, T.S.; Pallisgaard, N.; Møller, M.B.; Hasselbalch, H.C. High prevalence of arterial thrombosis in JAK2 mutated essential thrombocythaemia: independence of the V617F allele burden. *Hematology* **2008**, *13*, 71-76.
54. Passamonti, F.; Elena, C.; Schnittger, S.; Skoda, R.C.; Green, A.R.; Girodon, F.; Kiladjian, J.-J.; McMullin, M.F.; Ruggeri, M.; Besses, C. Molecular and clinical features of the myeloproliferative neoplasm associated with JAK2 exon 12 mutations. *Blood* **2011**, *117*, 2813-2816.
55. Vannucchi, A.; Lasho, T.; Guglielmelli, P.; Biamonte, F.; Pardanani, A.; Pereira, A.; Finke, C.; Score, J.; Gangat, N.; Mannarelli, C. Mutations and prognosis in primary myelofibrosis. *Leukemia* **2013**, *27*, 1861-1869.
56. Randi, M.L.; Lombardi, A.M.; Scapin, M.; Tezza, F.; Scandellari, R.; Ruzzon, E.; Duner, E.; Fabris, F. Haemostatic proteins gene polymorphisms in patients with unusual vein thrombosis and Ph-myeloproliferative disorders. *Thromb. Haemostasis* **2007**, *98*, 702-704.
57. Dambrauskienė, R.; Gerbutavičius, R.; Ugenskienė, R.; Jankauskaitė, R.; Savukaitytė, A.; Šimoliūnienė, R.; Rudžianskienė, M.; Gerbutavičienė, R.; Juozaitytė, E. Genetic polymorphisms of hemostatic factors and thrombotic risk in non BCR-ABL myeloproliferative neoplasms: A pilot study. *Balkan J. Med. Genet.* **2017**, *20*, 35-42.
58. Jensen, M.K.; de Nully Brown, P.; Thorsen, S.; Hasselbalch, H.C. Frequent occurrence of anticardiolipin antibodies, Factor V Leiden mutation, and perturbed endothelial function in chronic myeloproliferative disorders. *Am. J. Hematol.* **2002**, *69*, 185-191.
59. Buxhofer-Ausch, V.; Olcaydu, D.; Gisslinger, B.; Schalling, M.; Frantal, S.; Thiele, J.; Müllauer, L.; Kvasnicka, H.M.; Watzke, H.; Kralovics, R. Decanucleotide insertion polymorphism of F7 significantly influences the risk of thrombosis in patients with essential thrombocythemia. *Eur. J. Haematol.* **2014**, *93*, 103-111.
60. Maral, S.; Acar, M.; Balcik, O.S.; Uctepe, E.; Hatipoglu, O.F.; Akdeniz, D.; Altun, H.U.; Kosar, A.; Gunduz, M.; Gunduz, E. Matrix metalloproteinases 2 and 9 polymorphism in patients with myeloproliferative diseases: a STROBE-compliant observational study. *Medicine (Baltimore)* **2015**, *94*, 1-8.
61. Horvat, I.; Antolić, M.R.; Rončević, P.; Serventi-Seiwerth, R.; Zadro, R. Thrombosis in MPN patients and gene frequency of human platelet antigens. In Proceedings of MPN & MPNr-EuroNet Tenth Meeting *"Biology of sporadic and Hereditary Myeloproliferative Disease"*, Zagreb, Croatia; pp. 50-50.
62. Papadakis, E.; Papageorgiou, V.; Tsepanis, K.; Theocharidou, D.; Papadopoulos, V.K.; Georgiou, E.; Efraimidou, S.; Kioumi, A. Impact of Inherited Thrombophilia Factors On Thrombotic Risk in Patients with Newly Diagnosed BCR-Abl (-) Myeloproliferative Disorders; Finally a Role of MTHFR-C677T Polymorphism? *Blood* **2012**, *120*, 5065.
63. Krahling, T.; Balassa, K.; Kiss, K.P.; Bors, A.; Batai, A.; Halm, G.; Egyed, M.; Fekete, S.; Remenyi, P.; Masszi, T. Co-occurrence of myeloproliferative neoplasms and solid tumors is attributed to a synergism between cytoreductive therapy and the common TERT polymorphism rs2736100. *Cancer Epidemiol. Biomarkers Prev.* **2016**, *25*, 98-104.
64. Gorukmez, O.; Sag, Ş.O.; Gorukmez, Ö.; Ture, M.; Topak, A.; Sahinturk, S.; Ozkaya, G.; Gulten, T.; Ali, R.; Yakut, T. Association of the ACE I/D gene polymorphisms with JAK2V617F-positive polycythemia vera and essential thrombocythemia. *Genet. Test. Mol. Biomarkers* **2015**, *19*, 303-308.
